# Highly efficient removal of Sb(V) from water by franklinite-containing nano-FeZn composites

**DOI:** 10.1038/s41598-021-95520-7

**Published:** 2021-08-24

**Authors:** Huiqing Wu, Qingping Wu, Jumei Zhang, Qihui Gu, Weipeng Guo, Shun Rong, Yongxiong Zhang, Xianhu Wei, Lei Wei, Ming Sun, Aimei Li, Xinhui Jing

**Affiliations:** 1 Guangdong Provincial Key Laboratory of Microbial Safety and Health, State Key Laboratory of Applied Microbiology Southern China, Institute of Microbiology, Guangdong Academy of Sciences, Guangzhou, 510070 People’s Republic of China; 2 Guangdong Dinghu Mountain Spring Company Limited, Zhaoqing City, 526070 Guangdong Province People’s Republic of China

**Keywords:** Chemical biology, Environmental sciences, Anatomy, Chemistry, Engineering, Nanoscience and technology

## Abstract

The existence of toxic and carcinogenic pentavalent antimony in water is a great safety problem. In order to remove antimony(V) from water, the purpose of this study was to prepare a novel graphene nano iron zinc (rGO/NZV-FeZn) photocatalyst via hydrothermal method followed by ultrasonication. Herein, weakly magnetic nano-Fe–Zn materials (NZV-FeZn, GAC^SP^/NZV-FeZn, and rGO/NZV-FeZn) capable of rapid and efficient Sb(V) adsorption from water were prepared and characterised. In particular, rGO/NZV-FeZn was shown to comprise franklinite, Fe^0^, and graphite. Adsorption data were fitted by a quasi-second-order kinetic equation and Langmuir model, revealing that among these materials, NZV-FeZn exhibited the best Sb removal performance (543.9 mg_Sb_ g_NZV-FeZn_^−1^, *R*^2^ = 0.951). In a practical decontamination test, Sb removal efficiency of 99.38% was obtained for a reaction column filled with 3.5 g of rGO/NZV-FeZn. Column regenerability was tested at an initial concentration of 0.8111 mg_Sb_ L^−1^, and the treated water obtained after five consecutive runs complied with the GB5749-2006 requirement for Sb. rGO/NZV-FeZn was suggested to remove Sb(V) through adsorption-photocatalytic reduction and flocculation sedimentation mechanisms and, in view of its high cost performance, stability, and upscalable synthesis, was concluded to hold great promise for source water and wastewater treatment.

## Introduction

Water pollution is caused by the direct or indirect entry of untreated contaminants. When the pollution level exceeds the limits prescribed by the World Health Organisation and the United States Pharmacopoeia, the pollutants enter the food chain through bioaccumulation and cause serious harm to humans. As a typical heavy metal, Sb is toxic and carcinogenic to humans and other organisms, causing liver, skin, respiratory, and cardiovascular diseases^1^. Consequently, the United States Environmental Protection Agency and the European Union have prioritised the control of this pollutant^2^. China hosts more than half of the global Sb reserves and is accounted for 100% of the global Sb output from 1998 to 2008 (1.5 × 10^5^ tonnes)^3^. Consequently, Sb pollution is a very serious problem in China. In nature, Sb occurs in rocks, water, and soil at levels of 0.15–2 mg kg^−1^, < 1 mg mL^−1^, and 0.3–8.6 mg kg^−1^, respectively^4^, whereas in the Qingfeng River near the world’s largest Sb mine in the Xikuangshan area, the recorded values was as high as 6384 mg L^−1^^5–7^. The concentration of dissolved Sb in surface water near mining areas in China ranges from 4.58 to 29.4 μg L^−1^^8,9^. In the Yangtze River, Sb(III) and Sb(V) concentrations were determined as 0.029–0.736 and 0.121–2.567 mg L^−1^, respectively^7^, i.e. well above the maximal values stipulated by drinking water standards (e.g. a threshold of 0.005 mg L^−1^ is set by the GB5749-2006 national standard). Consequently, in order to be used for drinking, such water needs to be treated.

Currently, (electro)chemical (sulphide precipitation, pH adjustment), physicochemical (coagulation, ion exchange, and adsorption), and biological methods are used for the primary treatment of Sb mine wastewater^10–12^. In view of the toxicity of various Sb forms, biological treatment is inefficient and has not been extensively studied, with notable examples being green marine macroalgae and sulphate-reducing bacteria used for the decontamination of Sb-polluted water^13–16^. Conversely, physicochemical methods are more efficient and have been widely investigated, as exemplified by (electro)chemical oxidation^17^, electrocoagulation^18,19^, and adsorption^20–25^. Traditional Sb adsorbents include activated carbons, biochar, graphene, clays, and abundant minerals and cheap materials that have been successfully used for decades to remove toxic heavy metals from aqueous solutions. The high adsorption capacities and fast adsorption rates of nanomaterials and other popular new adsorbents, such as zero-valent nano-Fe, binary oxides, and metal–organic frameworks, make them well suited for the treatment of Sb-contaminated water^26–29^. Magnetic adsorbents such as MNP@hematite can be removed from water under the action of a magnetic field, thus offering an important advantage^30^. MIL-101(Fe) was reported to exhibit high Sb(III) and Sb(V) adsorption capacities (151.8 and 472.8 mg g^−1^, respectively), exceeding those of most known adsorbents^18^. However, the efficiency of Sb-contaminated water treatment remains unsatisfactory, as the methods capable of meeting drinking water standards have certain shortcomings, such as the use of magnetic materials, which may result in secondary pollution. Even in the case of combined membrane filtration methods, when most of the waste is removed in a magnetic field after treatment^31^, the membrane is easily polluted and should be frequently replaced, which increases treatment cost^32^. Additionally, electrocoagulation-based treatment requires large early-stage investments^19^.

Herein, weakly magnetic nano-Fe–Zn composites, including those with initially purified graphene as a carrier (rGO/NZV-Fe–Zn), novel biologically activated carbon as a carrier (GAC^SP^/NZV-Fe–Zn), and no carrier (NZV-Fe–Zn), were synthesised for the treatment of Sb-contaminated water and characterised by several instrumental techniques to determine their key properties and to shed light on the Sb(V) removal mechanism. The Sb removal efficiencies of these materials were tested under batch and continuous treatment conditions, and the kinetics/thermodynamics of Sb(V) removal and the related factors of influence (e.g. auxiliaries, pH, oxidants, light, oxygen, and temperature) were studied. Several Sb(V) treatment reactors were constructed with rGO/NZV-FeZn to remove the residual adsorbent from the treated water, thereby increasing water quality and decreasing treatment cost. The regeneration process was optimised, and the performance of the optimal treatment column was tested to demonstrate the practical applicability of the developed method and its compliance with the national hygienic standard for drinking water (GB5749-2006). Additionally, the stabilities of NZV-FeZn and rGO/NZV-FeZn were tested at different temperatures and illumination conditions to confirm the effectiveness of these materials for water treatment.

## Results

### Adsorbent synthesis

**Figure 1:**
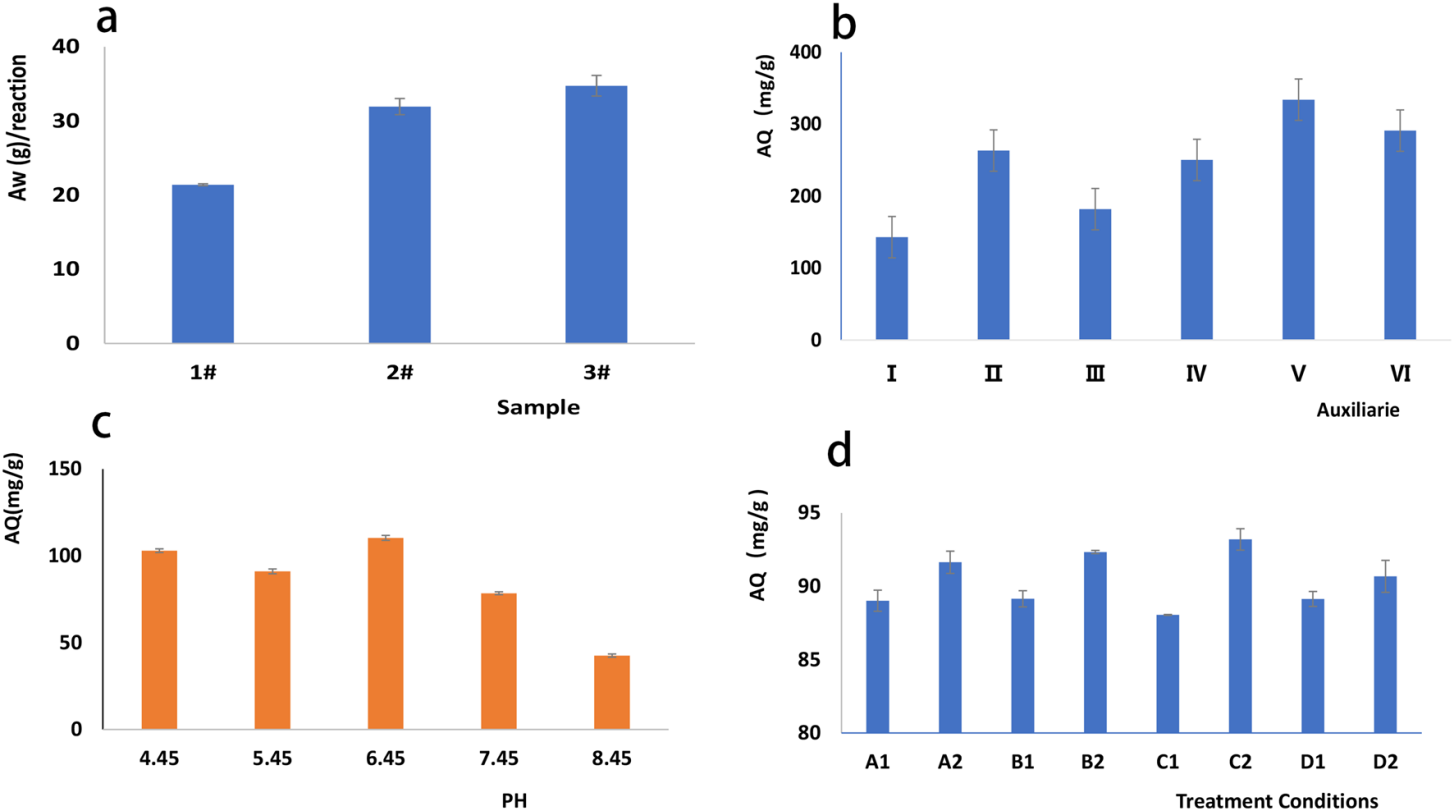
(**a**) Yields of NZV-FeZn (1#), rGO/NZV-FeZn (2#), and GAC^sp/^NZV-FeZn (3#) per unit mass. (**b**) Effects of auxiliaries on the efficiency of Sb(V) removal by rGO/NZV-FeZn. (**c**) Effect of pH on the efficiency of Sb(V) removal by rGO/NZV-FeZn. (**d**) Effects of illumination and oxygen on the efficiency of Sb(V) removal by rGO/NZV-FeZn.

On average, 21.53 g of NZV-FeZn (1#), 31.94 g of GAC^SP^/NZV-FeZn (2#), and 34.08 g of rGO/NZV-FeZn (3#) were obtained from 0 or 10 g of the carrier (rGO or GAC^SP^, 100 mesh) and other raw materials (Fig. 1a).

Ferrite nanomaterials are mainly prepared by hydrothermal and alkali treatment methods combined with high-temperature reduction^33,34^. In our search for optimal adsorbent preparation, we investigated the effects of alkali treatment and high-temperature calcination on adsorbent performance. Additionally, other technologies for the removal of Sb(V) from water were tested, such as Fe^2+^-mediated oxidation–reduction and treatment with pure FeSO_4_ and ZnSO_4_ as flocculants. Compared to the previously reported methods, the optimised technique presented herein offers the benefits of larger adsorbent yield and better Sb(V) removal efficiency (see Supporting Information). Moreover, the employed synthetic procedures are simple and easily upscalable, while the required raw materials (except for NaBH_4_ and graphene) are not expensive. Still, graphene can be prepared by the relatively inexpensive method of cooling-coupled ball milling, while NaBH_4_ can be replaced by other reducing agents, and activated carbon powder with a cheaper special formula can replace the synthesised materials on a large application scale. Therefore, the developed method allows excellent Sb sorbents to be prepared at a relatively low cost.

### Batch processing experiments

#### Effects of auxiliaries on Sb(V) removal efficiency

Figure 1b presents the effects of five auxiliaries, including HCOONa, white polymeric aluminium chloride (PAC-02), and anionic polyacrylamide (PAM), on the efficiency of Sb(V) removal by GAC^SP^/NZV-FeZn, revealing that this efficiency decreased in the following order: V > VI > II > IV > III > I. However, considering that raw water may contain other ions and organic substances/pigments, PAC-02 and PAM additives were concluded to be well suited for water treatment.

#### Effect of pH on Sb(V) removal efficiency

pH strongly affected the efficiency of Sb(V) removal by rGO/NZV-FeZn (Fig. 1c), which was ascribed to the decrease in Sb(V) solubility under alkaline conditions; the optimal pH was determined as 6.45.

#### Effects of illumination and oxygen on Sb(V) removal efficiency

Figure 1d presents the effects of illumination and oxygen on the efficiency of Sb(V) removal, revealing that for short treatment times, the effects of anaerobic + dark conditions were small and similar to those of anaerobic + light conditions. However, for a longer treatment time of two days, the best and second best effects were observed for anaerobic + dark and oxygen + light conditions, respectively. At this point, one should mention that the conditions were not strictly anaerobic or dark, as small amounts of oxygen and light were still present. For example, under dark conditions, the sample was covered by a box to block sunlight, i.e. it was still exposed to a certain amount of scattered light. Moreover, exposure to light occurred during sampling.

#### Adsorbent regenerability testing

**Figure 2:**
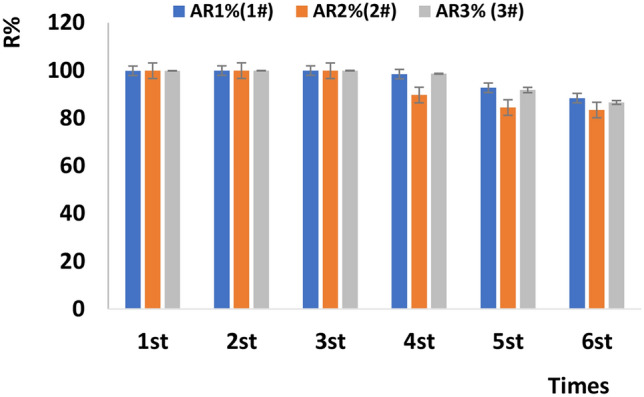
Results of adsorbent regenerability testing.

The batch adsorption process was repeated six times at an adsorbent loading of 10 g L^−1^ and initial Sb concentration of 9.173 mg L^−1^, with each set of experiments carried out in triplicate. The evaluated adsorbents performed well during the first five runs (Fig. 2).

#### Sorption isotherms and kinetics

**Figure 3:**
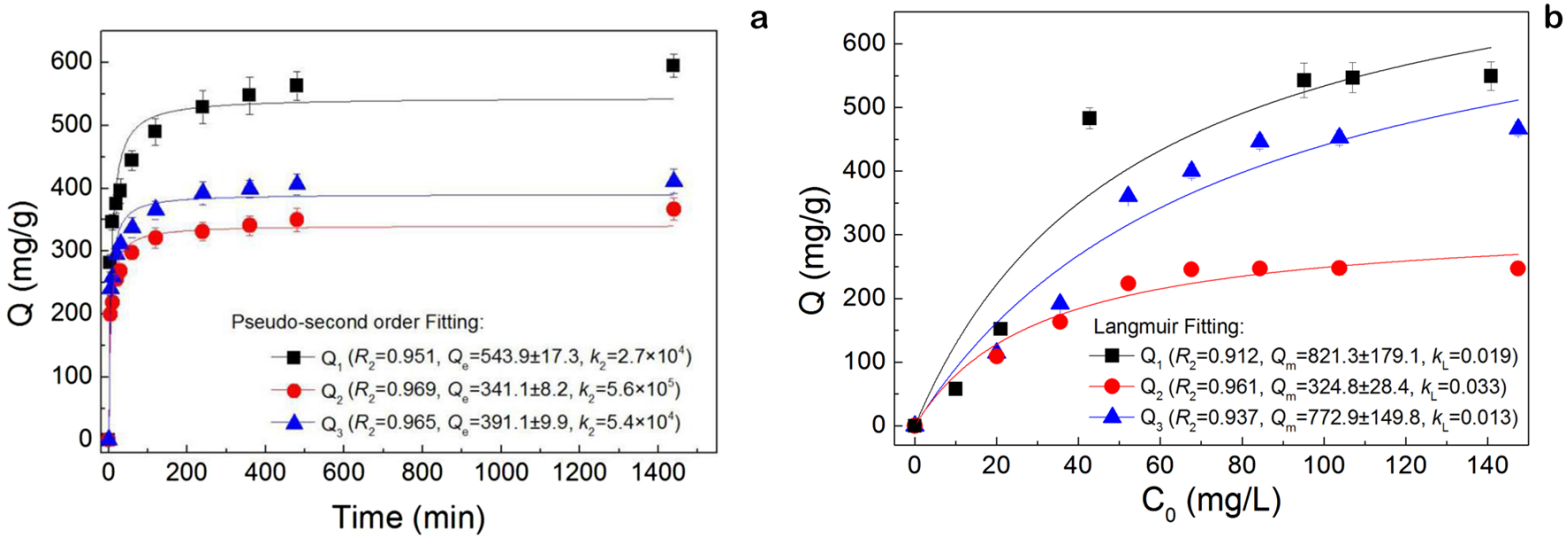
(**a**) Sorption kinetics and (**b**) isotherms obtained at adsorbent loadings of 0.05–0.1 g L^−1^ and initial Sb concentrations of 0–160 mg L^−1^.

Figure 3a,b present Sb(V) sorption kinetics and isotherms, revealing that Sb(V) removal was best described by a quasi-second-order kinetic model. In comparison, the Langmuir model was a more suitable thermodynamic model, as the reliability of the thermodynamic equation is not as good as that of the dynamic equation (evaluated in terms of *R*^2^). The maximum Sb(V) adsorption capacities of NZV-FeZn, GAC^SP^/NZV-FeZn, and rGO/NZV-FeZn were determined as 543.9, 341.1, and 391.1 mg g^−1^ (*R*^2^ = 0.951, 0.969, and 0.965), respectively.

Although the adsorption capacity of GAC^SP^/NZV-Fe–Zn was lower than that of rGO/NZV-FeZn, the former adsorbent was better suited for industrial-scale production, as GAC^SP^ is significantly cheaper than rGO.

### Continuous processing experiments

**Figure 4:**
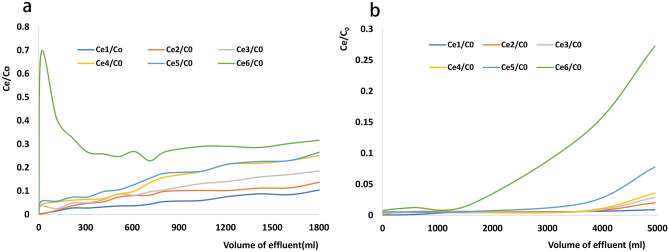
Results of regenerability testing obtained for columns with (**a**) 0.3 g of rGO/NZV-FeZn at an initial Sb concentration of 0.9324 mg L^−1^ and (**b**) 3.5 g of rGO/NZV-FeZn at an initial Sb concentration of 0.8111 mg L^−1^.

The results of column regenerability evaluation are presented in Fig. 4 and Table 1. Specifically, Fig. 4 presents the performances of columns with (a) 0.3 g of rGO/NZV-FeZn at an initial Sb concentration of 0.9324 mg L^−1^ and (b) 3.5 g of rGO/NZV-FeZn at an initial Sb concentration of 0.8111 mg L^−1^. Table 1 presents the contents of Fe, Zn, Al, and Sb in the effluents collected during six-fold treatment for columns with an adsorbent loading of 3.5 g.

**Table 1:** Fe, Zn, Al, and Sb contents in effluents collected after multiple runs.

Run no.	*V* (mL)	Sb (ppb)	Fe (ppb)	Zn (ppb)	Al (ppb)	*C*_e_/*C*_0_ (Sb)
1	3725	4.836	6.933	4.354	23.975	0.0060
2	3725	4.993	39.989	295.470	230.654	0.0062
3	3725	5.036	10.905	687.158	224.346	0.0062
4	3300	5.045	66.502	242.005	548.869	0.0062
5	1605	5.024	87.646	9.610	879.700	0.0062
6	15	6.041	88.160	12.255	692.524	0.0074

For the column in Fig. 4a, the effect of the first five treatments was better than that of the sixth treatment. In this case, the lower adsorbent loading and higher initial concentration of Sb resulted in a relatively low volume of effluent satisfying the condition of [Sb] = 0.005 mg L^−1^ (i.e. *C*_e_/*C*_0_ = 0.00536). However, if *C*_e_/*C*_0_ is set to 0.1055 (i.e. [Sb] = 0.09837 mg L^−1^(Actual measured value) and Sb removal efficiency = 83.4%), the column performance decreases only slightly during the first five runs, with the respective effluent volumes satisfying this condition determined as 1800, 1200, 800, 600, and 500 mL, respectively.

For the column in Fig. 4b, good performance was observed for all six runs. As the inflection point was set to 0.005 mg_Sb_ L^−1^, the effluents collected during the first five runs met the requirements of GB5749-2006. The corresponding Sb removal efficiency equalled 99.38%, and the volumes of the first five effluents satisfying this condition equalled 3725, 3725, 3725, 3300, and 1600 mL, respectively. However, after five-fold regeneration, the requirement of 0.005 mg L^−1^ could not be met. This result is consistent with that obtained for the column in Fig. 4a under batch conditions (Fig. 2). As the maximal allowable Sb level in drinking water is very low (0.005 mg L^−1^ according to GB5749-2006), the adsorbent amount should exceed 0.3 g to comply with this guideline.

Fe, Zn, Al, and other ions in the effluent of the column with an adsorbent loading of 3.5 g were monitored by ICP-MS to determine the suitability of the chosen material for drinking water purification. Table 1 shows that the effluents of the first five runs before the inflection point met the GB5749-2006 requirement for Sb, which corresponded to an Sb removal efficiency of 99.38%. The levels of Fe, Zn, and Al in the effluent collected during the second run did not comply with the GB5749-2006 stipulated values of 0.3, 1.0, and 0.2 mg L^−1^, respectively. Given that Fe, Zn, and Al are washed off the regeneration column during operation, the adopted method is suitable for drinking water treatment, which is in line with the use of powdered materials to remove pollutants from tap water. The unused recycled materials can be utilised for wastewater treatment to increase the material utilisation efficiency and decrease treatment costs.

### Effects of temperature and sunlight on adsorbent stability

**Figure 5:**
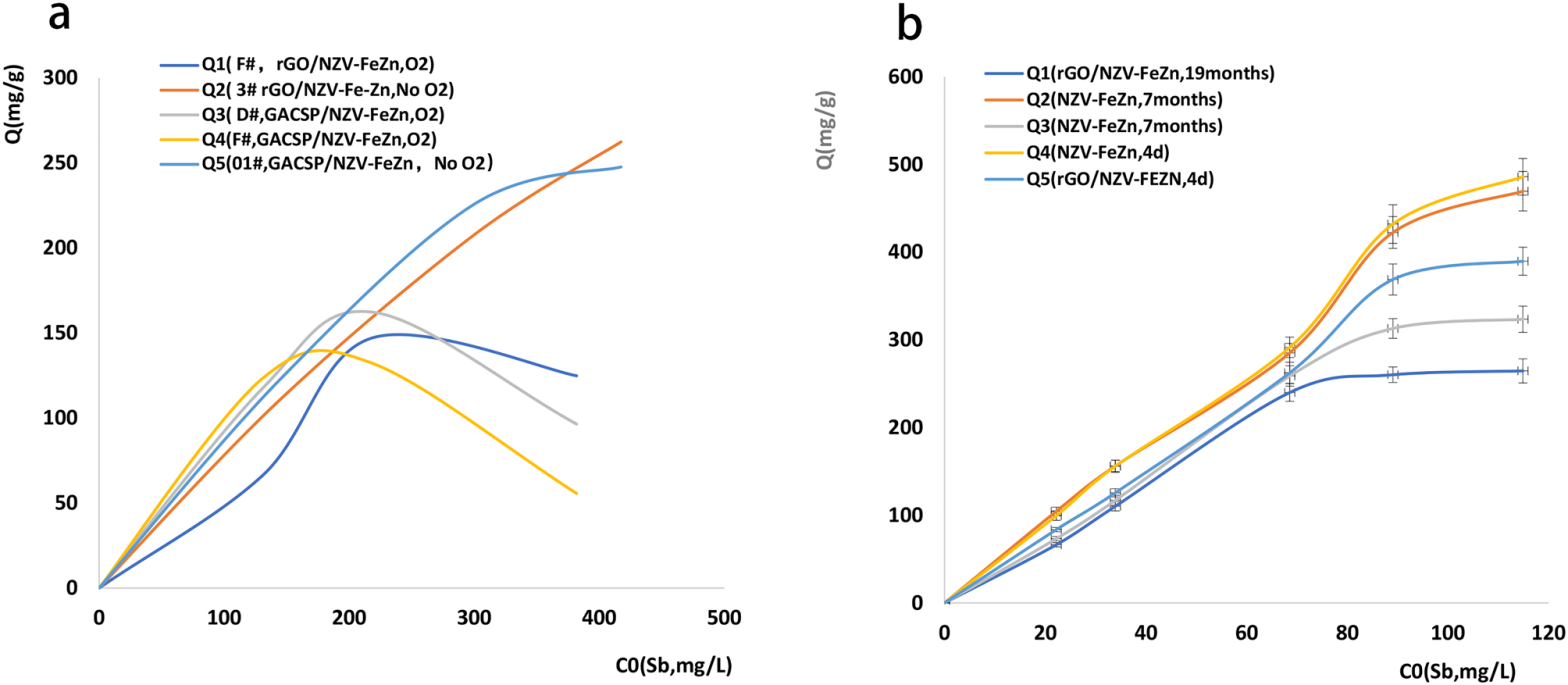
Effects of storage time, temperature, and sunlight on the Sb(V) removal performance of the selected adsorbents. Results obtained for (**a**) room temperature (20 °C), no sunlight, loading = 0.05 or 0.1 g L^−1^, adsorbent = NZV-FeZn or rGO/NZV-FeZn; (**b**) room temperature (20 °C), sunlight (measured temperature = 26 °C), loading = 0.1 g L^−1^, adsorbent = rGO/NZV-FeZn (2018042803) stored for 19 months (1#), NZV-FeZn (2019042801) stored for 7 months (2#), rGO/NZV-FeZn (2019042803) stored for 7 months (3#), freshly synthesised (4 days) NZV-FeZn (2019111401) (4#), and freshly synthesised (4 days) rGO/NZV-FeZn (2019111402) (5#).

The effects of storage time, temperature, and sunlight on the Sb(V) removal performance of the selected adsorbents are presented in Fig. 5.

The maximum treatment capacities of freshly synthesised NZV-FeZn (4#) and rGO/NZV-FeZn (5#) were approximately 380 and 270 mg g^−1^, respectively, and were both observed at an initial Sb concentration of 131.18 mg L^−1^. For sealed and dried NZV-FeZn stored for 7 months (2#) and rGO/NZV-FeZn stored for 7 months (3#), the maximal adsorption capacities equalled 258.2 and 245.8 mg g^−1^, respectively, and were observed at initial Sb concentrations of 95.56 and 68.18 mg L^−1^, respectively. The above values were lower (by 32.05 and 8.96%, respectively) than those of freshly synthesised materials (4# and 5#). The maximum adsorption capacity of rGO/NZV-FeZn stored for 19 months (1#) equalled 124.4 mg g^−1^ at an initial Sb concentration of 50.36 mg L^−1^, i.e. was lower than that of freshly synthesised rGO/NZV-FeZn (5#) by 67.26%. At the time of certification, the shelf life of nano-sized Fe and Zn and the materials loaded on graphene is more than 7 months, whereas the shelf life of nano-sized Fe and Zn loaded on graphene is longer.

Under the conditions of sunlight, room temperature (20–26 °C), and an adsorbent loading of 0.1 g L^−1^, the maximum adsorption capacity (486.04 mg g^−1^) of freshly prepared NZV-FeZn (4#) decreased by < 5% after 7-month storage (2#). In contrast, the maximum adsorption capacity (389.74 mg g^−1^) of freshly synthesised rGO/NZV-FeZn (5#) decreased by ~ 17 and 32% after 7- (3#) and 19-month (1#) storage, respectively. However, at lower initial loadings, these materials retained their capacity for 19 months.

Thus, if NZV-FeZn and rGO/NZV-FeZn are stored in sealed containers in a dry nitrogen atmosphere and tested in the absence of sunlight at 20 °C, the storage period is at least 7 months. The adsorption capacity of rGO/NZV-FeZn stored for 19 months was only ~ 33% of that of the freshly prepared material. When NZV-FeZn and rGO/NZV-FeZn were tested under sunlight at 20–26 °C, their Sb adsorption capacities decreased by < 5% after 7-month storage. Notably, these materials could be preserved for 19 months under the above optimal conditions (dry oxygen-free atmosphere, sunlight) and retailed 66% of their capacity at low initial loadings.

### Characterisation

#### Scanning electron microscopy (SEM) and transmission electron microscopy (TEM) analyses

**Figure 6:**
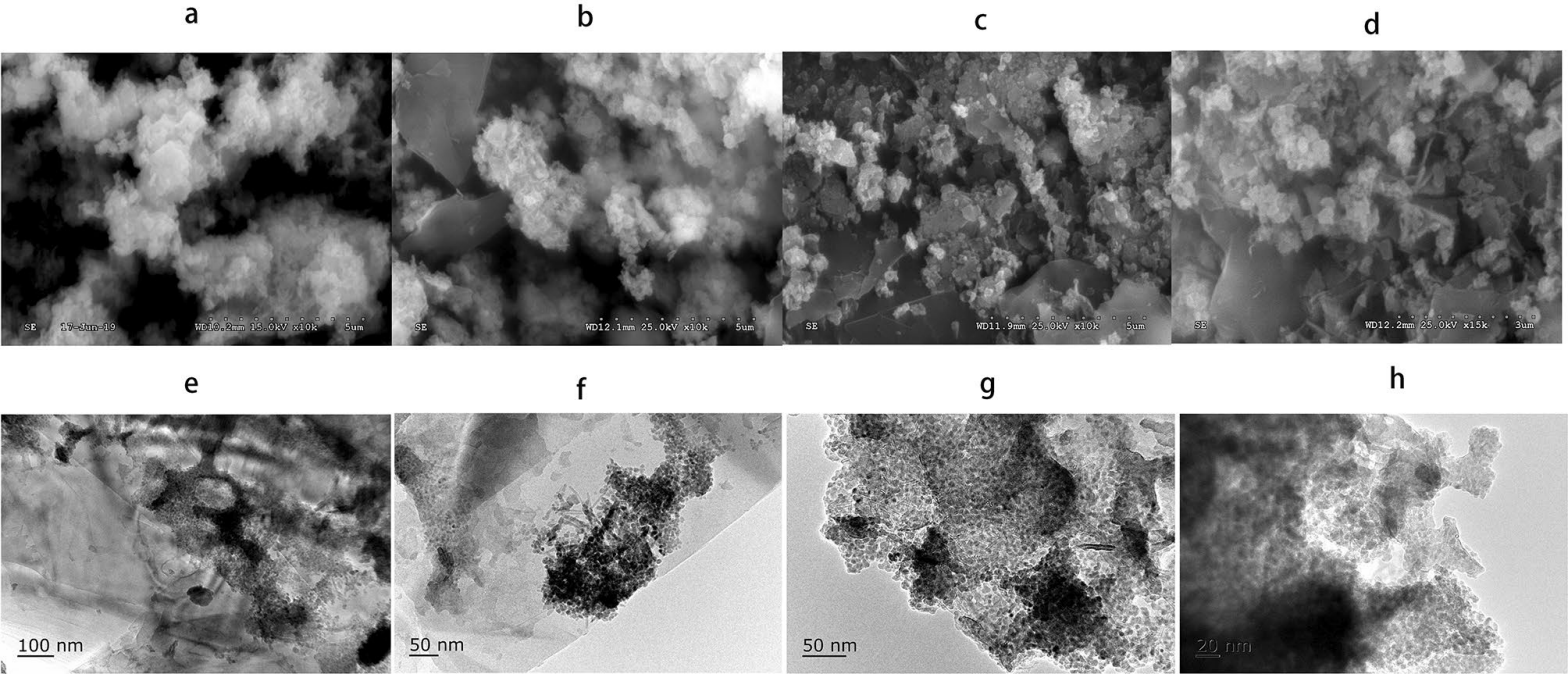
(**a–d**) SEM images of NZV-FeZn, rGO/NZV-FeZn, and rGO/NZV-FeZn(Sb) with micrograph resolution of 5 and 3 μm. (**e–h**) TEM images of rGO/NZV-FeZn with particle sizes of 200, 100, 100, and 50 nm.

SEM were performed with the synthetic nanoscale iron and zinc particles (including NZV-FeZn(2019111401), rGO/NZV-FeZn(2019111402)) and the used material(rGO/NZV-FeZn(Sb,2019111402)). Herein, Fig. 6a–d is the SEM images with micrograph resolution of 5 μm of NZV-FeZn(2019111401), 5 μm and 3 μm of rGO/NZV-FeZn(2019111402), 3 μm of rGO/NZV-feZn(Sb,2019111402). Figure 6e–h is the TEM images with micrograph resolution of 100,50,5 and 20 nm of rGO/NZV-FeZn (2019111402). From the SEM images, NZV-FeZn and rGO/NZV-FeZn all contain relatively small amounts of a flocculent substance. But, the used material of rGO/NZV-FeZn(Sb) showed that many particles of Fe Zn nanoparticles changed a little and the small particles increased after photocatalysis and low temperaturee drying. In addition, a large number of thin film like flakes and particles with 10–20 nm were observed in the TEM images of rGO/NZV-FeZn (Fig. 6e–h). However, the SEM results of the used material of rGO/NZV-FeZn(Sb) showed that the particles of Fe Zn nanoparticles changed a little and the small particles increased after photocatalysis and low temperature drying.

#### High-resolution TEM (HR-TEM) analysis and elemental mapping

**Figure 7:**
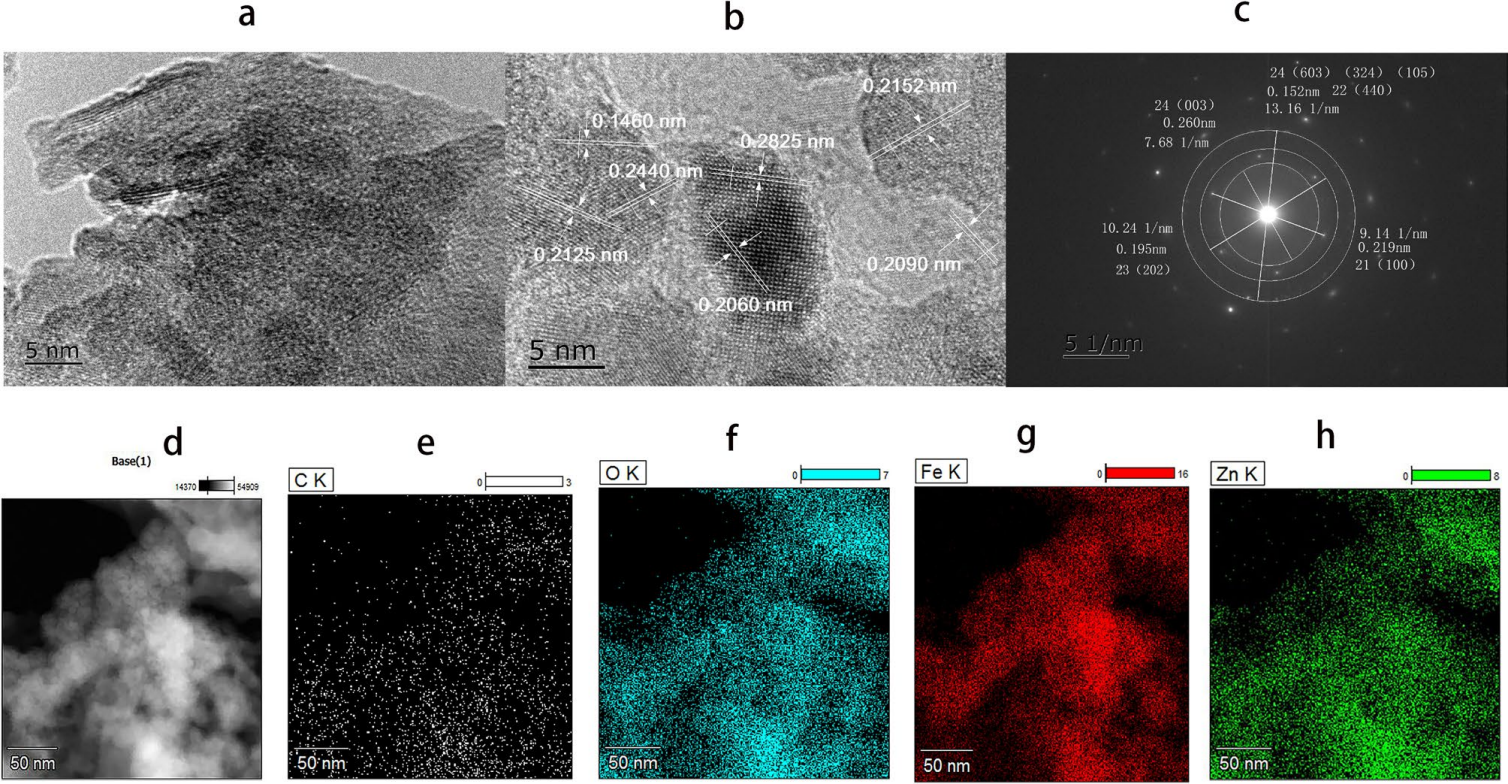
(**a–c**) HR-TEM images of rGO/NZV-FeZn with micrograph resolution of 5 and 2 nm and SAED pattern, (**d–h**) distribution maps of C, O, Fe, and Zn in rGO/NZV-FeZn.

**Figure 8:**
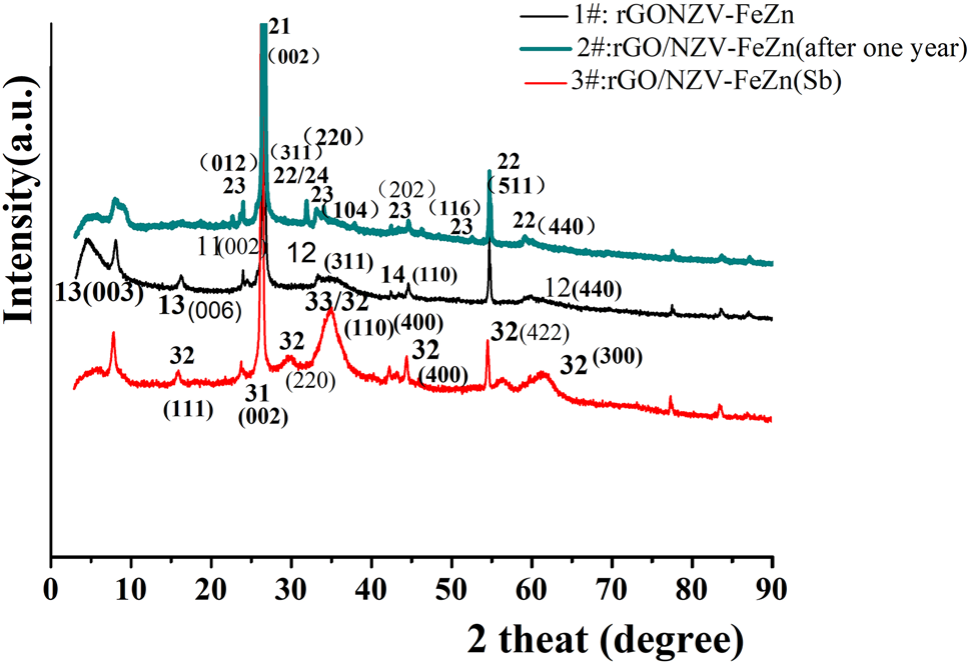
XRD patterns for rGO/ NZV-FeZn (1#: 20180091803, 2#: rGO/NZVFeZn (2019111402) and 3#: rGO/NZV-FeZn(Sb, 2019111402)).

The adsorbent crystal structure was further probed by HR-TEM and elemental mapping. Figure 7 shows high resolution TEM images (a, b) and SAED pattern o of rGO/NZV-FeZn (c) images and the EDX-mapping of the base, C, O, Fe and Zn element in the material of rGO/NZV-FEZn (d–h). Code of crystal material in synthetic materials and the crystal surface number (hkl) marked on high resolution TEM images and SAED pattern of rGO/NZV-FeZn also agreed well with the XRD results. It is evident from the TEM image images of 5 nm in Fig. 7a that they are many long strips of membranous and many small particles about 6–19 nm. HR-TEM image of rGO/NZV-FeZn (Fig. 7a–c) corroborated the successful formation of rGO/NZV-FeZn nanopowders, by depicting the presence of fringes having distance of 0.15, 0.21(0.2060, 0.2090, 0.2125, 0.2152),0.24 and 0.28 nm, corresponding to lattice spacing of cubic crystal structure of Fe_2.021_O_4_Zn_0.969_, hexagonal crystal structure of graphite-2H and FeSO_3_, and rhombohedral crystal structure of Fe_2_O_3_. SAED pattern also validated the formation of crystalline nanoparticles (Fig. 7c). From the element mapping of HR-TEM, it is known that the primary elements in rGO-NZV-FeZn are carbon, oxygen, iron, and zinc. A typical energy -dispersive X-ray spectroscopy mapping (EDX-mapping) image of the composite (Fig. 7d–h) agreed well with the XRD results (Fig. 8).

#### XRD

The crystalline properties and composites of the three samples including the new material (1#) and stored material for one year (2#) of rGO/NZV-FeZn and the used material (3#) of rGO/NZV-FeZn(Sb) (which was treated by photocatalysis and air drying at low temperature ) were studied using XRD method. Figure 8 shows XRD patterns for rGO/ NZV-FeZn (new), rGO/NZV-FeZn (after one year) and the used material of rGO/NZV-FeZn(Sb), and the numbers in brackets on the curve represent the crystal surface number (hkl) of XRD characterization material. The primary crystallite size of NZVs-FeZn microspheres were determined from the strongest XRD peak by Scherrer's formula^35^:

1 $${\text{D}} = 0.9\lambda /\beta \cos \theta$$

Based on comparisons to a database of XRD samples, the results suggest that the crystalline material in the XRD characterisation results show that sample of 1# contains graphite-2H(11, C, 30 ± 3A), franklinite, syn (12, (Zn0.803 Fe0.197) (Fe1.824 Zn0.166) O4, 32.6 ± 0.5A,22, graphite(13,C, 408 ± 10.8A), and iron (14, Fe,0.000); sample 2# contains graphite-2H(21, C, 30 ± 3A), franklinite, syn (22, (Zn_0.803_ Fe_0.197_) (Fe_1.824_ Zn_0.166_) O_4_, 23 ± 3A,) Hematite, syn(23, Fe2 O3, 33 ± 6A) and iron sulfite (24,FeSO_3_,26.5 ± 3A). Sample 3# contains Graphite-2H( 31, C, 34 ± 6)A),Franklinite (32, (Zn, Mn, Fe) (Fe, Mn)_2_ O_4_, 5.38 ± 8A) and Hematite, syn (33, Fe_2_ O_3,_ 37 ± 6A). By comparing the newly synthesized materials with the materials stored at room temperature for one year, it is found that the composition of the material has a little change. The zero valent iron in the old material (2#) is missing from the XRD characterization, but addition of ferrous sulfite. In addition, compared with untreated materials(2#), XRD result shows that composition of the used material (3#) of rGO/NZV-FeZn(Sb) had some changes. In which, zero valent iron basically disappears, part of divalent iron becomes trivalent iron, and the molecular formula of nano iron and zinc also had some changes, but the structure of nano iron and zinc has not been changed, the graphite crystal in the treatment process has also become smaller, and the reducibility is reduced in the used material of rGO/NZV-FeZn(Sb).

#### XPS analysis

**Figure 9:**
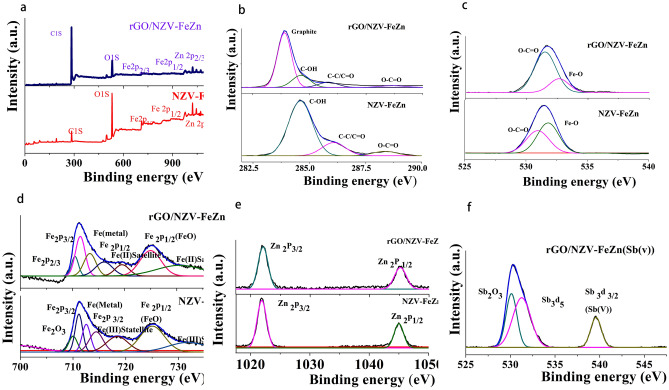
(**a**) X-ray photoelectron survey spectra of NZV-FeZn and rGO/NZV-FeZn, high-resolution (**b**) C 1s, (**c**) O 1s, (**d**) Fe 2p, and (**e**) Zn 2p spectra of NZV-FeZn and rGO/NZV-FeZn; **f,** high-resolution O 1s spectra of rGO/NZV-FeZn after Sb(V) via the photocatalytic reaction.

The valence states of elements in NZV-FeZn (2019111401), rGO/NZV-FeZn (2019111402) and the used material of rGO/NZV-FeZn(Sb, 2019111402) were probed by XPS (Fig. 9 and Table 2), which confirmed the presence of C, O, Zn, and Fe in NZV-FeZn and rGO/NZV-FeZn (Fig. 9a). The high-resolution C 1s spectrum of NZV-FeZn showed three peaks centred at 284.61, 286.09, and 288.36 eV, which were assigned to C–OH (adventitious carbon), C–C/C=C, and O–C=O, respectively (Fig. 9b). Interestingly, the spectrum of rGO/NZV-FeZn featured peaks at similar binding energies of 283.91, 284.88, 286.08, and 288.90 eV, which were assigned to graphite, C–OH, C–C/C=C, and O–C=O, respectively^36^. The high-resolution O 1s spectrum of NZV-FeZn (Fig. 9c) featured two peaks at 531.76 and 532.65 eV due to Fe–O and O–C=O, respectively, whereas in the O 1s spectrum of rGO/NZV-FeZn, these peaks were observed at 530.66 and 532.19 eV, respectively^37^. The Fe 2p spectrum of NZV-FeZn (Fig. 9d) featured seven peaks at 710.53, 711.82, 713.53, 716.35, 719.70, 724.95, and 730.19 eV, which were attributed to Fe_2_O_3_, Fe 2p_3/2_ (FeOOH), Fe 2p_3/2_ (Fe metal), Fe 2p_3/2_ (Fe^3+^, oxide) , Fe(III) (Fe 2p_3/2_, satellite), Fe 2p_1/2_ (FeO), and Fe(III) (Fe 2p_3/2_, satellite), respectively. Similarly, the Fe 2p spectrum of rGO/NZV-FeZn featured peaks at 703.27, 709.96, 711.41, 713.22, 725.05, and 729.76 eV, which were attributed to Fe 2p_3/2_ (metal), Fe 2p_3/2_ (metal), Fe 2p_1/2_(FeOOH or FeO), Fe (II) (Fe 2p_1/2_, satellite), Fe 2p_1/2_ (FeOOH or FeO), and Fe (II) (Fe 2p_1/2_, satellite)^37,38^ and were less intense than those of NZV-FeZn. Moreover, high-resolution Zn 2p spectra of NZV-FeZn and rGO/NZV-FeZn were recorded (Fig. 9e). In the case of NZV-FeZn, two sharp peaks at 1025.92 and 1045.03 eV were observed, corresponding to Zn 2p_3/2_ and Zn 2p_1/2_ transitions, respectively. In the case of rGO/NZV-FeZn, these peaks were observed at lower binding energies (1025.10 and 1045.2 eV, respectively), which indicated that, Zn existed as Zn^2+^ in the two adsorbents. These results suggested the transfer of electrons from NZV-FeZn to rGO. Finally, Fig. 9f is the high-resolution O 1s spectra of rGO/NZV-FeZn after Sb(V) featured three peaks at 530.11, 531.26, and 539.59 eV due to Sb(III) (Sb_2_O_3_), Fe–O/Sb(III)O3d_5/2_, and Sb 3d_3/2_ (Sb(V))^38,39^, respectively, which indicated that Sb(V) adsorbed by rGO/NZV-FeZn can be reduced to Sb(III). The XPS results (Fig. 9, Table 2) showed that rGO/NZV-FeZn largely comprised Zn^2+^-containing ferrite as well as Fe^0^ and Fe^2+^, whereas NZV-FeZn contained more Fe^3+^ and zinc ferrite, in line with XRD data.

**Table 2:** XPS characterization test results.

Element	Binding energy(eV)	Valence states of corresponding elements	Sample
C1s	284.61	C–OH	NZV-FeZn(2019111401)
	286.09	C–C/C=C	
	288.36	O–C=O	
O1s	530.66	Fe–O	
	531.19	O–C=O	
Fe2p	710.53	Fe_2_O_3_	
	711.82	Fe2p_3/2_ (FeOOH)	
	713.53	Fe2p_3/2_(Fe, metal)	
	716.35	Fe2p_3/2_(Fe^3+^, Oxidesate)	
	719.7	Fe2p_2/3_(Fe(III), Satellite)	
	724.95	Fe2p_1/2_ (FeO)	
	730.19	Fe2p_1/2_(Fe(III), Satellite)	
Zn2p	1025.9	Zn 2p _3/2_	
	1045.3	Zn2p_1/2_	
C1s	283.91	Graphite	rGO/NZV-FeZn (2019111402)
	284.88	C–OH	
	286.08	C–C/C=C	
	288.9	O–C=O	
O 1s	531.76	O–C=O	
	532.65	Fe–O	
Fe2p	703.27	Fe2p_3/2_ (metal)	
	709.96	Fe2p_3/2_(metal)	
	711.41	Fe2p_3/2_ (FeOOH or FeO)	
	713.22	Fe2p_3/2_(Fe, metal)	
	725.05	Fe2p_1/2_ (FeO)	
	729.97	Fe2p_1/2_(Fe(II), Satellite)	
Zn2p	1025.1	Zn 2p_3/2_	
	1045.2	Zn2p_1/2_	
Sb3dO1s	530.13	Sb_2_O_3_	rGO/NZV-FeZn(Sb, 2019111402)
	531.59	Fe–O/Sb(III)O3d_5/2_	
	539.59	Sb3d_3/2_(Sb(V))	

#### Electrochemical characterisation of rGO, NZV-FeZn, and rGO/NZV-FeZn

**Figure 10:**
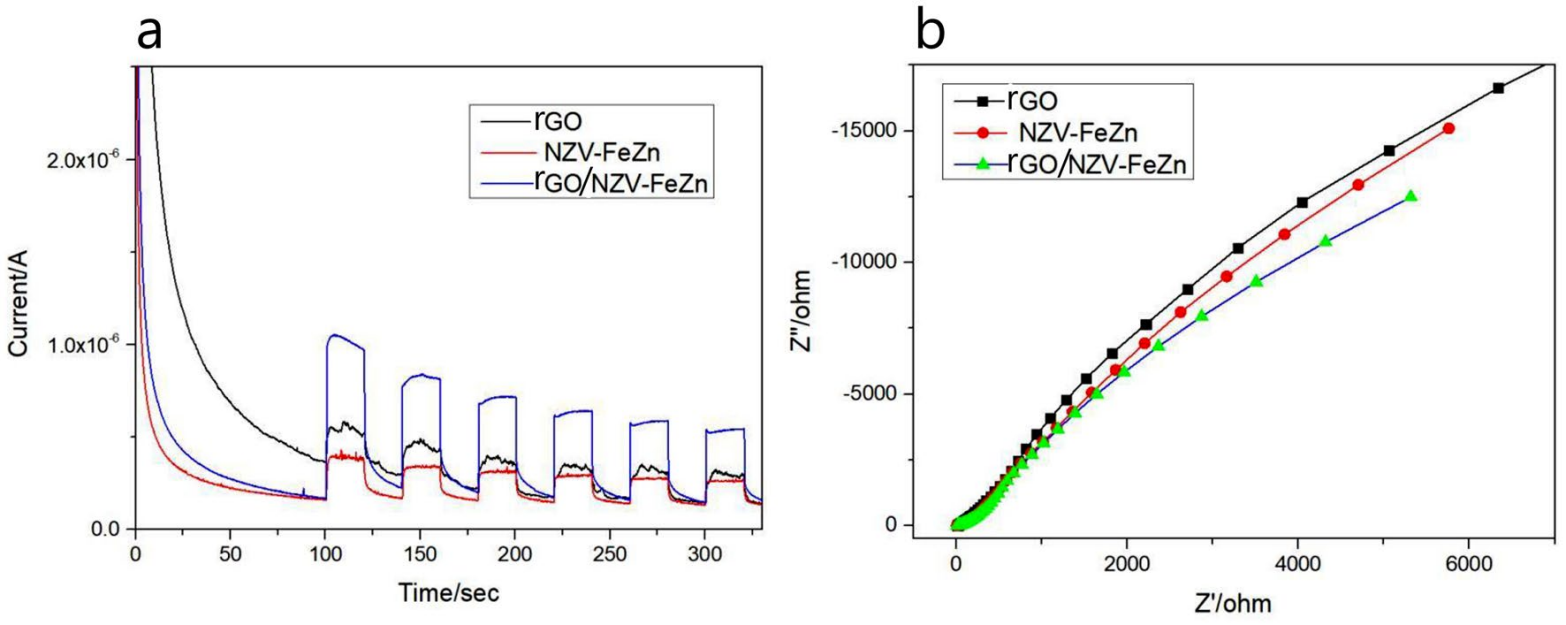
(**a**) Photocurrent response curves recorded under full-wavelength artificial light irradiation and (**b**) Nyquist plots of rGO, NZV-FeZn, and rGO/NZV-FeZn.

Further insights into the photocatalytic properties of rGO, NZV-FeZn, and rGO/NZV-FeZn were provided by electrochemical characterisation. Specifically, transient photocurrent response and electrochemical impedance spectroscopy (EIS) photocurrent measurements were performed to probe the efficiency of electron–hole separation. Figure 10a shows the transient photocurrent responses of rGO, NZV-FeZn, and rGO/NZV-FeZn in several light cycles under full-wavelength artificial light irradiation. Under the same conditions, the photocurrent response intensities of bare rGO and NZV-FeZn were significantly lower than that of rGO/NZV-FeZn, which indicated that the heterojunction constructed with rGO/NZV-FeZn can promote the separation of photogenerated electrons and holes. Figure 10b presents the Nyquist plots (real impedance (*Z*) vs. imaginary impedance (*Z″*’)) of rGO, NZV-FeZn, and rGO/NZV-FeZn obtained under full-wavelength artificial light irradiation. Generally, charge transfer efficiency is negatively correlated with arc radius. This radius was smaller for rGO/NZV-FeZn than that for NZV-FeZn, which indicated that charge transfer was faster in the former case. Thus, the heterojunction formed between rGO and NZV-FeZn can significantly promote the separation and transfer of photogenerated charges, thereby extending their lifetime. Overall, all three tested materials exhibited good electrochemical properties^40^.

#### UV–vis diffuse reflectance spectroscopy and PL spectra of the three samples

**Figure 11:**
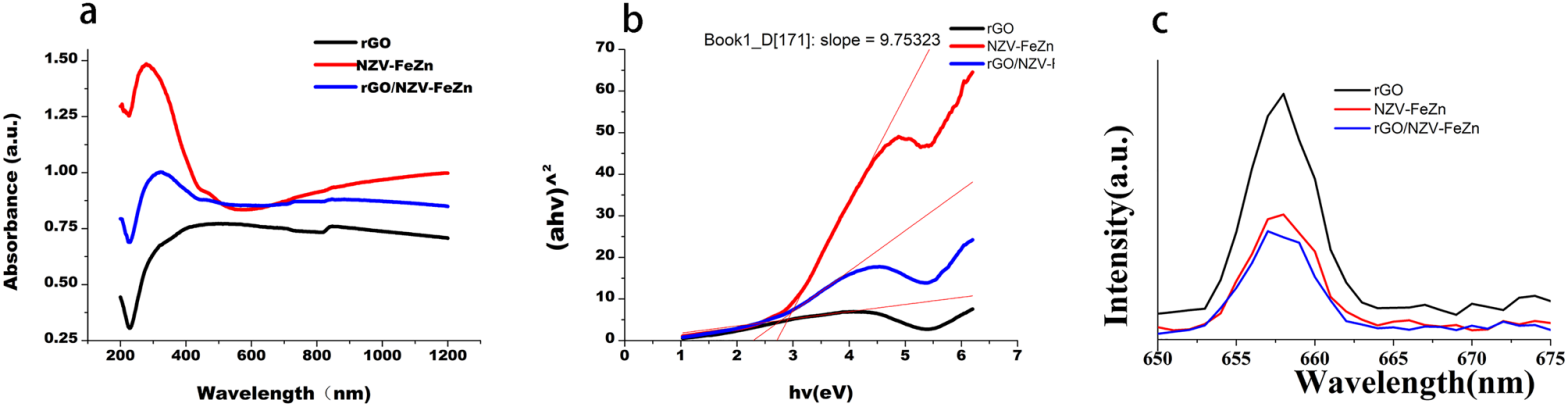
(**a**) Figure 11. a, UV-vis diffuse reflectance spectra ,b, (ahν)2-hν curves and c, Pl of rGO, NZV-FeZn, and rGO/NZV-FeZn. UV–vis diffuse reflectance spectra and (**b**) (*ahν*)^2^-*hν* curves of rGO, NZV-FeZn, and rGO/NZV-FeZn.

Figure 11a presents the UV–vis diffuse reflectance spectra of rGO, NZV-FeZn, and rGO/NZV-FeZn. Since graphene, as well as NZV-FeZn and rGO/NZV- FeZn, were conductive, their spectral data were processed according to conductor type. Pure NZV-FeZn had an obvious visible light absorption edge at ~ 630 nm. Interestingly, enhanced absorption was observed within the range of 800–1200 nm, and rGO exhibited a broad absorption peak (200–1200 nm) due to light scattering by black graphite^41^. rGO/NZV-FeZn showed a relatively smooth and broad absorption peak at 400–1200 nm. Compared with that of NZV-FeZn, the spectra of rGO and rGO/NZV-FeZn became smoother at absorption edges, which was attributed to light scattering by rGO. The absorption edge of rGO/NZV-FeZn was located at 848 nm (Fig. 11a), which showed that the loading of rGO on NZV-FeZn resulted in absorption edge broadening and enhanced the absorption capacity and utilisation efficiency of visible light. The band gaps (*E*_g_) of rGO, NZV-FeZn, and rGO\NZV-FeZn were determined as 0, 2.68, and 2.2 eV, respectively from (*ahν*)^2^ versus *hν* plots (Fig. 11b). This indicates that hybridisation with rGO changed the energy-band structure of NZV-FeZn, i.e. reduced the band gap. Fig.13c presented the PL emission spectra of rGO, NZV-FeZn, and rGO/NZV-FeZn. The PL spectrum could reflect the recombination rate of photogenerated electron and hole in photocatalyst. The lower PL intensity indicated that the separation effect of photogenerated electron hole pair is better. The major emission wavelength of samples was located at 657-658 nm when excited at 370 nm. Apparently, the photoluminescence spectra of rGO and NZV-FeZn displayed strong intensity, demonstrating low separation efficiency of photoelectron and hole pairs. More importantly, compared with rGO and NZV-FeZn, rGO/ NZV-FeZn showed a significant decrease of photoluminescence intensity, which proved that construction of composite materials promoted the separation of the photogenerated charges, and inhibited the electron and hole recombination, and finally improved the photocatalytic efficiency.

#### Energy-band properties

**Figure 12:**
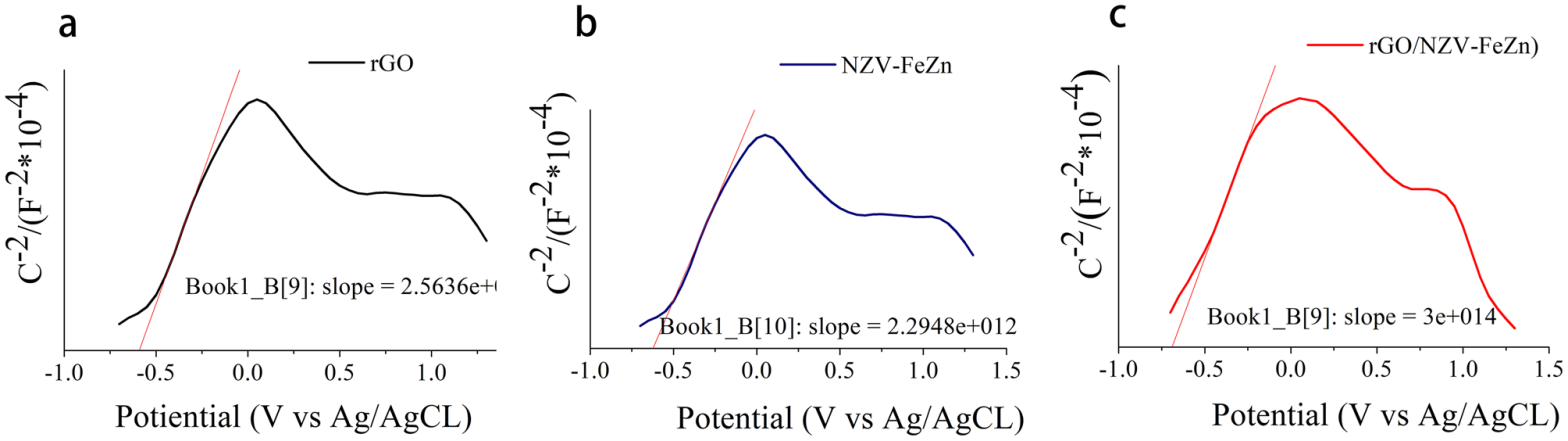
Mott–Schottky plots of (**a**) rGO, (**b**) NZV-FeZn, and (**c**) rGO/NZV-FeZn.

The redox capabilities of the photogenerated electrons and holes arriving at the semiconductor surface depend on the valence band (VB) and conduction band (CB) potentials, respectively. Therefore, Mott–Schottky plots are suitable for the analysis of flat-band potentials and were used to characterise the energy-band properties of the synthesised adsorbents (Fig. 12).

rGO, NZV-FeZn, and rGO/NZV-FeZn displayed distinct *n*- and *p*- type semiconductor characteristics^42^, and their flat-band potentials (*E*_CB_) were determined as − 0.625, − 0.625, and − 0.688 eV vs. Ag/AgCl, respectively, or as − 0.665, − 0.665, and − 0.733 eV vs. SCE, respectively. The VB potentials of rGO, NZV-FeZn, and rGO/NZV-FeZn were calculated as − 0.655, 2.025, and 1.467 eV vs. SCE, respectively, using the relationship *E*_VB_ = *E*_g_ − *E*_CB_^43^.

### Elucidation of the Sb(V) removal mechanism

#### Reductive flocculation hypothesis

**Figure 13:**
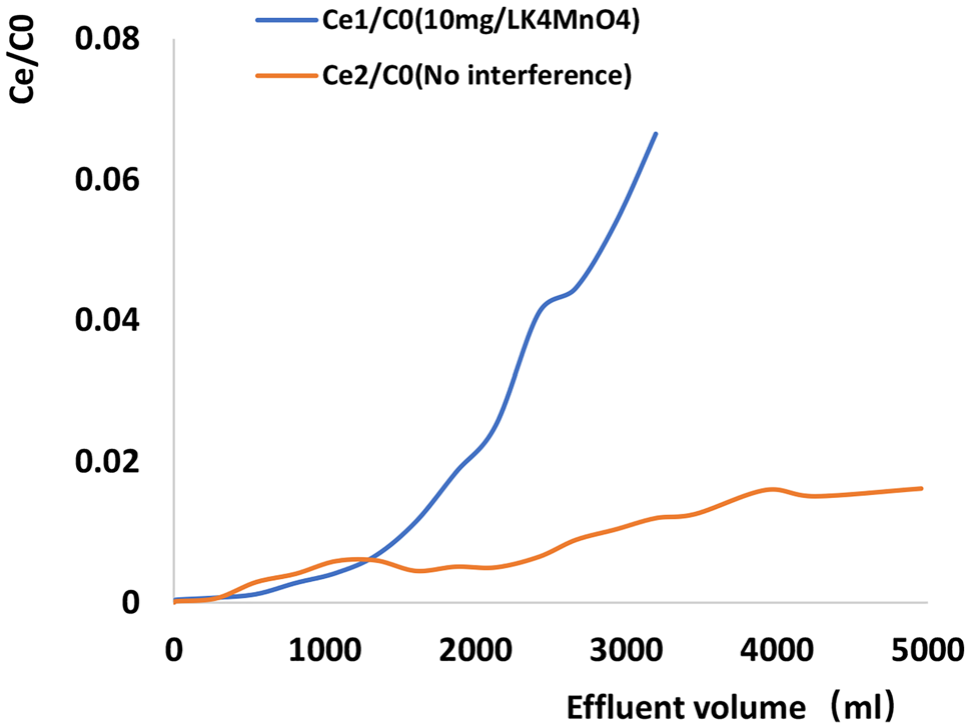
Removal efficiency of pentavalent antimony in water by the same 3.5-g rGO/NZV-FeZn column under the pentavalent antimony water containing 10 mg/LKMNO_4_.

The efficiency of Sb(V) removal by a 3.5-g rGO/NZV-FeZn column significantly decreased in the presence of 10 mg L^−1^ KMnO_4_ (Fig. 13), as in this case, Mn was removed in preference to Sb. The concentration of Mn in the effluent was below the detection limit (1 ppb). This result, together with the fact that flocculants were present in Sb-contaminated water, suggested that the removal of Sb(V) proceeded via reductive flocculation according to the mechanism proposed by Wu et al. (2019) for the removal of Cr(VI)^44^.

#### Photocatalytic hypothesis

**Figure 14:**
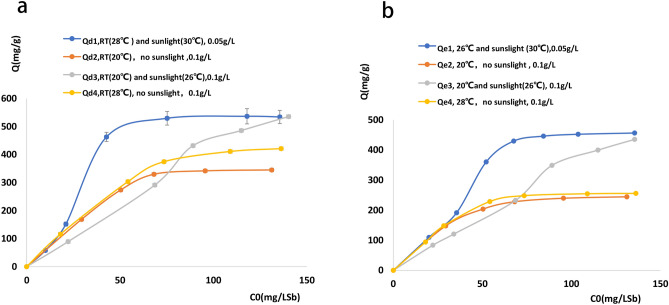
Effect of light, temperature, and dosage on removal of antimony pentavalent from water of the synthetic materials NZV-FEZn (**a**) and rGO/NZV-FeZn (**b**).

Figure 14 shows the Sb removal performances of freshly prepared rGO/NZV-FeZn and NZV-FeZn at different temperatures and variable illumination conditions.

At an adsorbent loading of 0.1 g L^−1^, the Sb removal performance of NZV-FeZn (a1) and rGO/NZV-FeZn (b1) could be enhanced using high initial Sb concentrations, temperature of 20 °C, and sunlight (the measured temperature equalled 26 °C (a3 and b3)). A dynamic balance was reached at 20 °C in the absence of sunlight at a loading of 0.1 g L^−1^ (a2 and b2) or at 26 °C in the presence of sunlight (30 °C) at a loading of 0.05 g L^−1^ (a1 and b1). However, at an ambient temperature of 26 °C in the presence of sunlight (30 °C), the average temperature was calculated as 28 °C, and the maximum adsorption capacities of both materials (a1, b1) were much higher than those (a4, b4) obtained at 28 °C in the absence of sunlight. Therefore, sunlight was concluded to activate the removal of Sb by both adsorbents, i.e., this removal was photocatalytic in nature.

Photocatalysis allows solar energy to be directly converted into chemical energy and is therefore viewed as a green and sustainable solution to energy production and environmental problems. Temperature is usually a vital factor controlling the kinetics and thermodynamics of a given reaction. Menga et al*.* (2018) showed that the photocatalytic activity of three catalysts increased with increasing temperature^45^, in line with our results.

#### Physical adsorption

Subsequently, we probed the textural properties of adsorbents and correlated them with Sb removal efficiencies (Table 3).

**Table 3:** Selected textural properties of adsorbents.

Sample	*S*_BET_ (m^2^ g^−1^)	Zeta potential (mV)	Total Fe content (%)	Total Zn content (%)	Total pore volume (m^3^ g^−1^)
NZV-FeZn, 1#	36.899	− 24.4	33.88	20.90	0.017582
rGO, 2#	23.273	− 22.3	0	0	0.01287
rGO/NZV-FeZn, 3#	33.28	− 36.9	15.6	9.67	0.01287

All three adsorbents had high zeta potentials and specific surface areas, and Sb adsorption thereon was speculated to involve physisorption. Notably, hybridisation with rGO increased the specific surface area and the absolute zeta potential of Fe–Zn nanoparticles, i.e., provided more active sites and a higher reduction potential.

### Sb(V) removal mechanism by rGO/NZV-FeZn

**Figure 15:**
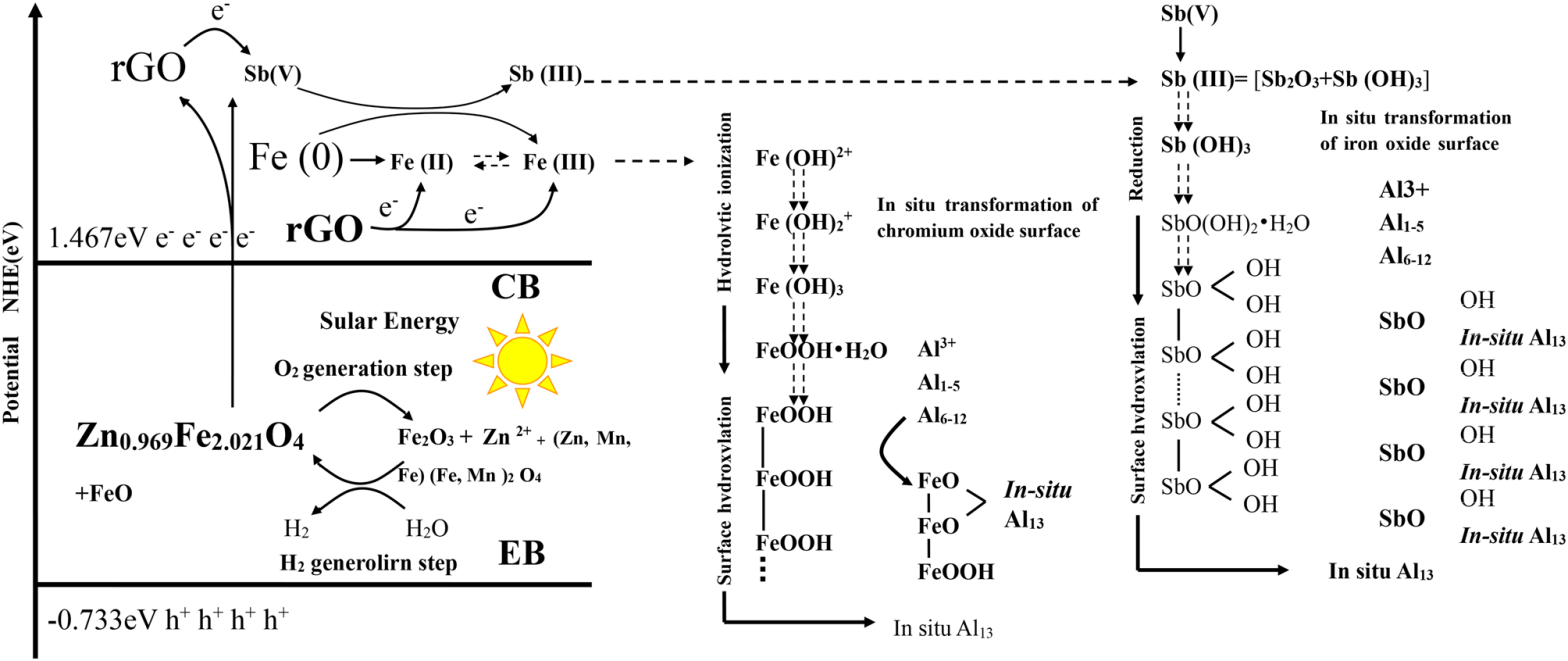
Schematic mechanism of Sb(V) removal by rGO/NZV-FeZn.

Figure 15 presents the adsorption-photocatalytic reduction and flocculation sedimentation mechanisms of Sb(V) removal by rGO/NZV-FeZn. The enhanced photocatalytic activity of this adsorbent was mainly attributed to its broad photoabsorption and the effective separation of photogenerated electron–hole pairs. The central catalytic component (NZV-FeZn) is excited under full-wavelength artificial light irradiation because of its narrow band gap (2.68 eV). The zero-valent Fe in the above composite is a strong reductant. Additionally, rGO can act as a reductant and provides a narrower band gap (2.2 eV). The removal of Sb(V) was assumed to primarily involve chemical reduction, physisorption, flocculation, and photocatalytic action, which was demonstrated experimentally. Therefore, the following mechanism was proposed. Irradiation of the semiconducting zinc ferrite ((Zn_0.803_ Fe_0.197_)(Fe_1.824_ Zn_0.166_)O_4_) affords electrons that reduce Sb(V) to Sb(III), with additional electrons provided by the conversion of Fe^0^ to Fe(II) and Fe(III). Sb(III) and Fe(III) ions react with water to afford the corresponding hydroxides, which precipitate on the adsorbent under the flocculating action of polyaluminum chloride. Finally, rGO is believed to accelerate the transfer of photoelectrons to Sb(V) because of its large specific surface area and good electrical conductivity.

## Discussion

Weakly magnetic NZV-FeZn, GAC^SP^/NZV-FeZn, and rGO/NZV-FeZn effectively and rapidly removed Sb(V) from water, and rGO/NZV-FeZn contained franklinite, zero-valent Fe, and graphite. The adsorption of Sb(V) was described using quasi-second-order and Langmuir models. Among the tested materials, the highest adsorption capacity of 543.9 mg_Sb_ g^−1^ was observed for NZV-FeZn (*R*^2^ = 0.951). However, rGO/NZV-FeZn was selected for continuous Sb(V) removal because of its higher yield and efficiency (see Supporting Information). At an initial Sb(V) concentration of 0.8111 mg L^−1^, the reaction column constructed with 3.5 g of rGO/NZV-FeZn exhibited good Sb removal efficiency (99.38%) and regenerability during the first five operations before the inflection point met the GB5749-2006. As the spent catalyst was not dispersed in the treated water, it was concluded to be safe, and further characterisation provided important insights into the mechanism of Sb(V) removal. Thus, rGO/NZV-FeZn was concluded to be a cheap, stable, and efficient material for Sb removal from source water or waste water on both small and large scales. XRD results showed that rGO/NZV-FeZn contains not only spinel ((Zn_0.803_ Fe_0.197_)(Fe_1.824_ Zn_0.166_) O_4_) but also a small amount of zero-valent Fe and graphene. The experimental results suggested that rGO/NZV-FeZn primarily removes Sb(V) through adsorption-photocatalytic reduction and flocculation sedimentation mechanisms.

## Methods

### Materials

All reagents were purchased from commercial sources and used without further purification. Sb–K tartrate (KSbC_4_H_4_O_7_·0.5H_2_O, CAS: 11071-15-1, MACKLIN, AR, *M*_W_ = 333.98) used to prepare Sb(V) solutions, K_2_Cr_2_O_7_, HCOONa, FeSO_4_·7H_2_O, ZnSO_4_·7H_2_O, PEG-6000, anhydrous ethanol, macroporous resin D-01, and others including hydrochloric acid (HCL) and sodium hydroxide (NaOH) were purchased from Guangzhou Shuo Chemical Reagent Co., Ltd. PAM (anionic number ≥ 30,000) was sourced from Guangzhou Fang Bo Environmental Protection Technology Co., Ltd., while PAC-02 (drinking water level, Al_2_O_3_ ≥ 29%) was provided by Guangzhou Jin Xin Chemical Co., Ltd. All solutions were prepared using Milli-Q deoxygenated ultrapure water (18 MΩ cm, Easy Pure’II RF/UV, USA). High-purity graphite was purchased from Qingdao Huatai Lubrication and Sealing Technology Co., Ltd. (Qingdao, Shandong, China), and high-purity nitrogen and argon were sourced from Dacheng (Guangzhou) Gas Co., Ltd., Guangzhou, China.

### Synthetic procedures

The preparation of GAC^SP^ and the synthesis of graphene from high-purity graphite (rGO) was performed following the method reported by Wu et al. (2019)^44^.

NZV-FeZn–based adsorbents were prepared by liquid-phase reduction as described elsewhere^44^. For comparison, unsupported pristine NZV-FeZn was synthesised without the addition of PPG or GAC^SP^ according to the following equation:

2 $$\begin{aligned} & 11{\text{Fe}}^{2 + } { + }5{\text{Zn}}^{2 + } + {\text{SO}}_{4}^{2 - } + 22{\text{BH}}_{4}^{ - } + 6{\text{H}}_{2} {\text{O}} + {\text{PEG}} - 6000 \\ & \quad \to \left( {{\text{Zn}}\left( {{\text{OH}}} \right)_{2} } \right)_{3} \left( {{\text{ZnSO}}_{4} } \right)\left( {{\text{H}}_{2} {\text{O}}} \right)_{3} + 3{\text{Fe}}_{2} {\text{O}}_{3} + {\text{C}}_{9} {\text{H}}_{12} {\text{O}}_{7} {\text{Zn}} \cdot {\text{H}}_{2} {\text{O}} \\ & \quad + \left( {{\text{Zn}}_{0.969} {\text{Fe}}_{0.024} } \right){\text{Fe}}_{1.997} {\text{O}}_{4} + 3{\text{Fe}} + 2{\text{B}}\left( {{\text{OH}}} \right)_{3} + 7{\text{H}}_{2} \\ \end{aligned}$$

The synthetic protocol was optimised as follows. A suspension of 100-mesh rGO or GAC^SP^ (0 or 10 g) in a degassed aqueous solution of 1.1 M FeSO_4_, 0.5 M ZnSO_4_, and 1% PEG-6000 (100 mL) was ultrasonicated at 80 kHz for 120 min. Subsequently, a freshly prepared solution of NaBH_4_ in degassed water/absolute ethanol (30:70, v/v; NaBH_4_:(Zn^2+^  + Fe^2+^) = 2:1, mol/mol) was added to the suspension at 50–60 drops min^−1^, and the mixture was allowed to react for 2 h. The solid was separated via centrifugation at 10000 rpm for 15 min, dispersed in 200-250 ml deionised water, and sedimented by centrifugation at 10000 rpm for 15 min. The rinsing-sedimentation procedure was performed one more time, and the solid was vacuum-dried in an oven and stored in sealed vials under argon for subsequent experiments. The following batch numbers were used: NZV-FeZn (Nos. 20190125B, 2019042801, 2019040501, 2019111401); GAC^SP^/NZV-FeZn (Nos. 2018073005, 20190125D, 20190125F, 20190125G, 2019042802, 2018091801); and rGO/NZV-FeZn (Nos. 2018091803, 2019042803, 2019111402).

### Batch processing experiments

#### Effect of auxiliaries on Sb(V) removal

rGO-NZV-FeZn (No. 2018091803) was used as a Sb removal agent at a loading of 1 g L^−1^. System I contained 2 mM Sb with no auxiliary; system II contained 2 mM Sb, 2 mM HCOONa, 10 mg L^−1^ PAC-02, and 0.5 mg L^−1^ PAM; system III contained 2 mM Sb and 2 mM HCOONa; system IV contained 2 mM Sb, 10 mg L^−1^ PAC-02, and 0.5 mg L^−1^ PAM; system V contained 2 mM Sb and 10 mg L^−1^ PAC-02; and system VI contained 2 mM Sb and 0.5 mg L^−1^ PAM.

#### Effect of pH on Sb(V) removal

The pH of original solution (2 mM Sb without auxiliaries) was adjusted to 4.51, 5.45, 6.49, 7.49, or 8.49 with 0.01 M HCl, 0.05 M NaOH, and ultrapure water, and was checked using a pH meter. The sample volume was adjusted to 20 mL, and the specimens were denoted as I–V. Each specimen (5 mL) was treated with 5.0 mg of rGO-NZV-FeZn (No. 2018091803) and left to stand for 22 h, centrifuged for 2 min at 12,000 rpm, diluted 40,000-fold, and probed by ICP-MS. All the experiments were performed in triplicate.

The effects on Sb(V) removal of sunlight and oxygen were probed using a 25-mL transparent and capped scaled centrifugal tube containing 10 mL of contaminated water with an initial Sb concentration of ~ 1 mM and an adsorbent loading of ~ 1 g L^−1^. First, 138.94 mg of NZV-FeZn (No. 201901801) was added upon mixing to 134 mL of the Sb solution with an initial Sb concentration of ~ 1 mM, and the suspension was divided into 10-mL portions that were placed into 25-mL centrifugal tubes for testing the effects of oxygen and sunlight. Each experiment was performed in triplicate. When the adsorbent was mixed with Sb-containing water, the vial was opened and immediately capped again. Then, the specimens were exposed to light or placed in the dark for 4 or 24 h, sampled, centrifuged, and probed by ICP-MS as described above. The following labels were used: open cap = O_2_^+^, closed cap = O_2_^−^, bright location = hν^+^, and dark location = dark. The treatment conditions were denoted as A1 (O_2_^−^, hν, 4 h), A2 (O_2_^−^, hν, 48 h), B1 (O_2_^+^, hν, 4 h), B2 (O_2_^+^, hν, 48 h), C1 (O_2_^+^, dark, 4 h), C2 (O_2_^+^, dark, 4 h), D1 (O_2_^+^, dark, 4 h), and D2 (O_2_^+^, dark, 48 h).

#### Regenerability testing

Adsorbents 1# (NZV-FeZn, 2019042801), 2# (GAC^SP^/NZV-FeZn, 2019042802), and 3# (rGO-NZV-FeZn, 2019042803) were used at a loading of 10 g L^−1^, and each experiment was performed in triplicate.

Batch processing was performed as follows. Sb-containing water (9.173 mg_Sb_ L^−1^, 25 mL) and the adsorbent of choice (250 mg) were mixed for 15 min at room temperature, and the suspensions were centrifuged at 10,000 rpm for 15 min. The supernatant was collected and analysed by ICP-MS. During regeneration, the precipitate obtained after centrifugation was treated with dilute hydrochloric acid (5 mL, 0.1 MHCL) for 30 min. Then, the mixture was supplemented with water (35 mL) and centrifuged for 10 min at 10,000 rpm for three times. The supernatant was collected and analysed by ICP-MS. The process was repeated six times.

#### Sorption isotherms and kinetics

Sb(V) removal was performed at adsorbent loadings of 0.05–1.0 g L^−1^ using NZV-FeZn (1#, 2019042801), GAC^SP^/NZV-FeZn (2#, 2019042802), and rGO/NZV-FeZn (3#, 2019042803). Sb(V)-polluted water with initial Sb concentrations of 0–420 mg L^−1^ was prepared from pure water and Sb-K tartrate. Typically, 50 mL of the Sb(V) solution and 5.0–50 mg of the adsorbent were mixed and reacted for 24 h at room temperature. Samples taken at certain time intervals were centrifuged at 12,600 rpm for 2 min and appropriately diluted for analysis by ICP-MS (Agilent 1260-7700e; Agilent Technologies Co. Ltd., USA). The adsorption capacities (or handling capacities; *q*_e_) and the total Sb removal efficiencies were calculated as follows:

3 $$q_{{\text{e}}} = \left( {C_{0} - C_{{\text{e}}} } \right)/X,$$

4 $${\text{Removal}}\;{\text{efficiency}}\;\left( \% \right) = 100 \, \times \left( {C_{0} - C_{{\text{e}}} } \right)/C_{0} ,$$

where *X* is the sorbent loading (g L^−1^), and *C*_0_ and *C*_e_ (mg L^−1^) are the initial and equilibrium concentrations of Sb(V), respectively.

Pseudo-first-order and pseudo-second-order models were used to characterise the kinetics of Sb(V) adsorption^46^. with the integrated forms of these models given as follows:

5 $$q_{t} = q_{{\text{e}}} \left[ {1 - \exp \left( { - k_{{\text{t}}} } \right)} \right],$$

6 $$q_{t} = t/\left[ {\left( {1/k_{2} q_{{\text{e}}}^{2} } \right) + \left( {t/q_{{\text{e}}} } \right)} \right],$$

where *q*_t_ is the Sb(V) adsorption capacity at time *t* (mg g^−1^), and *k*_1_ (s^−1^) and *k*_2_ (g mg^−1^ s^−1^) are the pseudo-first-order and pseudo-second-order rate constants, respectively.

The equilibrium and kinetics of Sb(V) adsorption were probed using batch experiments under optimised conditions (adsorbent loading = 0.1 g L^−1^, initial Sb(V) concentration = 124.47 mg L^−1^, 30 °C, agitation at 100 rpm, and pH 6.5) at different time intervals. Three common isotherm models, namely Langmuir (Eq. 7)^47^, Freundlich (Eq. 8)^48^, and Langmuir–Freundlich (Eq. 9)^49^ models were used to describe the adsorption equilibrium.

7 $$q_{{\text{e}}} = K_{{\text{L}}} q_{{\text{m}}} C_{{\text{e}}} /\left( {1 + K_{{\text{L}}} C_{{\text{e}}} } \right),$$

8 $$q_{{\text{e}}} = K_{{\text{F}}} C_{{\text{e}}}^{1/n} ,$$

9 $$q_{{\text{e}}} = q_{{\text{m}}} \left( {K_{{\text{L}}}^{{\prime }} C_{{\text{e}}} } \right)^{{n{\prime }}} /\left[ {\left( {1 + K_{{\text{L}}}^{{\prime }} C_{{\text{e}}} } \right)^{{n{\prime }}} } \right],$$

where *q*_m_ is the maximum adsorption capacity (mg g^−1^), *K*_L_ is the Langmuir adsorption equilibrium constant (L mg^−1^), *K*_F_ and *n* are Freundlich constants representing the adsorption capacity (L ^−1/n^ mg ^−(1−n)^ g ^−1^ ) and the adsorption intensity varying with the degree of heterogeneity, respectively, and *K*_L_’ and *n*’ are the Langmuir adsorption equilibrium constant (L mg^−1^) and the adsorption intensity, respectively^50^.

Adsorption isotherms were studied using initial Sb(V) concentrations of 0–140 mg L^−1^, adsorbent loadings of 0.05 g L^−1^ (NZV-FeZn) and 0.1 g L^−1^ (rGO/NZV-FeZn and GAC^SP^/NZV-FeZn), temperature of 30 °C, 100-rpm agitation, and pH 6.5. For kinetic experiments, the adsorbent was mixed with Sb-contaminated water, and the residual Sb concentration was measured at various time intervals for up to 24 h.

### Continuous processing experiments

To meet the requirement of GB 5749–2006 ([Sb] < 0.005 mg L^−1^), we probed the effects of reducing the initial concentration and increasing the adsorbent loading, revealing that the latter strategy is more effective than the former. Specifically, a glass column (3.5 cm in diameter and 60 cm in height) was packed with 0.3 or 3.5 g of rGO/NZV-FeZn (2019042803) and 35 g of K-04 (granular activated carbon for pure water sterilisation) to a fixed bed depth of ~ 15.0 cm using ~ 1.5 g (3 × 0.5 g) of cotton wool and 10.0 g of macroporous resin soaked in ethanol. Aqueous Sb(V) solutions were prepared with initial concentrations of 0.8111–0.9324 mg L^−1^ and contained PAC-02 (10 mg L^−1^) and PAM (0.5 mg L^−1^) as auxiliaries. During operation, ~ 5000 mL of Sb-contaminated water was passed through the column at a flow velocity of 9–10.0 mL min^−1^. The initial 35 mL of the effluent was discarded, and samples were subsequently collected every 200 mL (± 15 mL) and analysed by ICP-MS to determine the concentrations of Fe, Zn, Al, and Sb. During regeneration, the column was washed with 200 mL of pure water after each operation and then soaked in 50–100 mL of 0.1 M HCl for > 20 h. Column regenerability was evaluated in terms of the ratio of Sb (Ce/C0 < or = 0.2) level in the effluent water of different volumes and initial water at different moving times.

### Effects of temperature and sunlight on adsorbent stability

Samples stored for 4 days, 7 months, and 19 months were used as adsorbents at loadings of 0.05–0.1 g L^−1^ to treat Sb(V) solutions with concentrations of 0–140 mg L^−1^. Then, the Sb(V) removal performance and stability of rGO/NZV-FeZn and NZV-FeZn were evaluated at different temperatures and irradiation conditions. The employed materials and batch numbers (t) are as follows: 1#, rGO/NZV-FeZn (2018091803); 2#, NZV-FeZn (209042801); 3#, rGO/NZV-FeZn (2019042803); 4#, NZV-FeZn (2019111401); and 5#, rGO/NZV-FeZn (2019111402).

The control materials (NZV-FeZn and rGO/NZV-FeZn) were synthesised from the same raw materials at different times by the same method (except for rGO loading) and were placed in a compact bag that was evacuated in a desiccator and then filled with nitrogen. Sb(V) solutions containing the desiccant were prepared with pure water and Sb-K tartrate at initial concentrations of 0–140 mg L^−1^. The Sb(V) solution (50 or 100 mL) was treated with NZV-FeZn or rGO/NZV-FeZn (5.0 mg), and the mixture was allowed to react for 48 h at 20 °C in the presence or absence of light. Subsequently, the samples were centrifuged at 12,000 rpm for 2 min, and the concentrations of Sb(V) in the supernatants were determined by ICP-MS. The adsorption (or handling) capacity and the total Sb(V) removal efficiency were calculated as described above.

### Characterisation

The surface morphologies of NZV-FeZn, GAC^SP^/NZV-Fe–Zn, and rGO/NZV-FeZn were examined using SEM (JEOL Ltd., Japan), while particle sizes were determined using TEM (JEOL Ltd., Japan). TEM and HR-TEM images were acquired on a JEOL JEM-2100F instrument. XRD patterns were recorded on an Ultima IV diffractometer at a scan rate of 2° min^−1^ within a 2*θ* range of 10–90° using Cu K-beta radiation and an accelerating voltage of 40 kV. Brunauer–Emmett–Teller (BET) surface areas, zeta potentials, and total Fe contents were determined according to GB/T 19587-2017 (determination of specific surface area of solid substances by gas adsorption BET method), GB/T 32666-2016 (general rules for gel particle zeta electroanalysis by electrophoresis), and JT/T 015-1996 general principles of inductively coupled plasma atomic emission spectrometry), respectively. All electrochemical measurements were performed on a CHI 660E electrochemical workstation (Shanghai Chenhua Instrument Co. Ltd., China) using a three-electrode system at room temperature. The working electrode was prepared on fluoride-tin oxide (FTO) conductor glass that was cleaned by sequential sonication in chloroform, acetone, and ethanol (30 min for each solvent). Adsorbent powder (5.0 mg) was mixed with pure water (0.5 mL) upon 30-min sonication, and 0.1 mL of the resulting slurry was diluted to 2 mg mL^−1^ with pure water (0.3 mL) and 0.5 wt% Nafion (0.1 mL, CAS:66796-30-3,alfa). The diluted slurry was dropped onto FTO glass and dried at 60 °C to afford a working electrode with an exposed area of 1 cm^2^. A Pt plate and a Ag/AgCl (3 M KCl) electrode were used as counter and reference electrodes, respectively. Aqueous Na_2_SO_4_ (0.2 M) was used as the electrolyte. Mott–Schottky plots were constructed for the abovementioned three-electrode system using the impedance-potential technique. Element contents and valence states were investigated using XPS (ESCALAB 250xi). Diffuse reflectance spectra were recorded on a UV–vis-NIR scanning spectrophotometer (UV3600, Shimadzu) using an integrating sphere.

### Elucidation and verification of the Sb(V) removal mechanism

Based on the fast and efficient removal of Sb(V) by the tested adsorbents, we suggested that this removal occurs via reductive flocculation, photocatalysis, and physisorption, with further details provided below.

#### Reductive flocculation–based removal

Two Sb removal columns were loaded with 3.5 g of rGO/NZV-FeZn (2019042803) and 35 g of K-04 as described above. Sb-contaminated water (1.2–1.4 mg_Sb_ L^−1^) containing PAC-02 (10 mg L^−1^) and PAM (0.5 mg L^−1^) as auxiliaries was divided into two parts. One part was used directly, while the other was supplemented with KMnO_4_ (10 mg L^−1^). The flow rate was controlled at 9–10 mL min^−1^, and a comparative test was carried out. Sampling of 15 mL per tube at 250-mL intervals, 35 ml waste liquid was discharged before sampling. In addition to 100 times dilution of the original solution, other effluent samples were detected using ICP-MS with the original solution to test the Sb content.

#### Photocatalytic removal

Freshly synthesised NZV-FeZn (d, 2019111401) and rGO/NZV-FeZn (e, 2019111402) were used at loadings of 0.05–0.1 g L^−1^. Sb(V) solutions had initial concentrations of 0–140 mg L^−1^. The Sb(V) solution (50 or 100 mL) was mixed with the adsorbent (5.0 mg) and reacted for 48 h under different conditions. The same volume of Sb solution with a different initial concentration was placed in a clean glass bottle (150 mL) as a blank control. After 48 h, the samples were centrifuged at 12,000 rpm for 2 min, and the concentrations of Sb(V) in the supernatant were analysed by ICP-MS. The adsorption (or handling) capacity and the total Sb(V) removal efficiency were calculated as described above. Each experiment was performed in triplicate. The reaction conditions were as follows. A: Reaction temperature = 20 °C, no additional sunlight. B: Temperature in reaction chamber = 20 and 26 °C in the absence and presence of additional sunlight, respectively; in the absence of sunlight, the sample was returned to the chamber at 20 °C. C: Reaction temperature = 28 °C, no sunlight. D: Temperature in reaction chamber = 26 and 30 °C in the absence and presence of additional sunlight, respectively; when there was no sunlight (6:15 PM to 8:15 AM), the sample was returned to the chamber at 26 °C.

### Statistical analysis

Each set of batch sorption experiments was conducted in triplicate. One-way ANOVA with Dunnett’s post hoc test and Tukey’s multiple comparison test was conducted using GraphPad Prism 5.0 software to determine the statistical significance of the results.

## Supplementary Information

Supplementary Information.

## References

[1] Bagherifam S, Brown TC, Fellows CM, Naidu R (2019). Bioavailability of arsenic and antimony in terrestrial ecosystems: A review. Pedosphere.

[2] Pierart A, Shahid M, Séjalon-Delmas N, Dumat C (2015). Antimony bioavailability: Knowledge and research perspectives for sustainable agricultures. J. Hazard. Mater..

[3] He M, Wang X, Wu F, Fu Z (2012). Antimony pollution in China. Sci. Total Environ..

[4] Herath I, Vithanage M, Bundschuh J (2017). Antimony as a global dilemma: Geochemistry, mobility, fate and transport. Environ. Pollut..

[5] Wang X, He M, Xi J, Lu X (2011). Antimony distribution and mobility in rivers around the world’s largest antimony mine of Xikuangshan, Hunan Province, China. Microchem. J..

[6] Xu R (2019). Impacts of antimony and arsenic co-contamination on the river sedimentary microbial community in an antimony-contaminated river. Sci. Total Environ..

[7] Yao C, Jiang X, Che F, Wang K, Zhao L (2019). Antimony speciation and potential ecological risk of metal(loid)s in plain wetlands in the lower Yangtze River valley, China. Chemosphere.

[8] Li J (2018). Antimony contamination, consequences and removal techniques: A review. Ecotoxicol. Environ. Saf..

[9] Aksoy N, Şimşek C, Gunduz O (2009). Groundwater contamination mechanism in a geothermal field: A case study of Balcova, Turkey. J. Contam. Hydrol..

[10] Long X, Wang X, Guo X, He M (2020). A review of removal technology for antimony in aqueous solution. J. Environ. Sci..

[11] Carolin CF, Kumar PS, Saravanan A, Joshiba GJ, Naushad M (2017). Efficient techniques for the removal of toxic heavy metals from aquatic environment: A review. J. Environ. Chem. Eng..

[12] Hashim MA, Mukhopadhyay S, Sahu JN, Sengupta B (2011). Remediation technologies for heavy metal contaminated groundwater. J. Environ. Manage..

[13] Ungureanu G, Santos SCR, Volf I, Boaventura RAR, Botelho CMS (2017). Biosorption of antimony oxyanions by brown seaweeds: Batch and column studies. J. Environ. Chem. Eng..

[14] Filote C (2017). Green macroalgae from the Romanian coast of Black Sea: Physico-chemical characterization and future perspectives on their use as metal anions biosorbents. Process Saf. Environ. Prot..

[15] Zhang G, Ouyang X, Li H, Fu Z, Chen J (2016). Bioremoval of antimony from contaminated waters by a mixed batch culture of sulfate-reducing bacteria. Int. Biodeterior. Biodegrad..

[16] Zhang H, Hu X (2019). Bioadsorption and microbe-mediated reduction of Sb(V) by a marine bacterium in the presence of sulfite/thiosulfate and the mechanism study. Chem. Eng. J..

[17] Moreira FC, Boaventura RAR, Brillas E, Vilar VJP (2017). Electrochemical advanced oxidation processes: A review on their application to synthetic and real wastewaters. Appl. Catal. B.

[18] Zhu J, Wu F, Pan X, Guo J, Wen D (2011). Removal of antimony from antimony mine flotation wastewater by electrocoagulation with aluminum electrodes. J. Environ. Sci..

[19] Cao D, Guo T, Zhao X (2019). Treatment of Sb(V) and Co (II) containing wastewater by electrocoagulation and enhanced Sb(V) removal with Co(II) presence. Sep. Purif. Technol..

[20] Aguayo-Villarreal IA, Bonilla-Petriciolet A, Muñiz-Valencia R (2017). Preparation of activated carbons from pecan nutshell and their application in the antagonistic adsorption of heavy metal ions. J. Mol. Liq..

[21] Lahori AH (2017). Use of biochar as an amendment for remediation of heavy metal-contaminated soils: Prospects and challenges. Pedosphere.

[22] Nupearachchi CN, Mahatantila K, Vithanage M (2017). Application of graphene for decontamination of water; Implications for sorptive removal. Groundw. Sustain. Dev..

[23] Uddin MK (2017). A review on the adsorption of heavy metals by clay minerals, with special focus on the past decade. Chem. Eng. J..

[24] Eskandari E (2020). A review on polyaniline-based materials applications in heavy metals removal and catalytic processes. Sep. Purif. Technol..

[25] Yao S (2020). Simultaneous oxidation and removal of Sb (III) from water by using synthesized CTAB/MnFe_2_O_4_/MnO_2_ composite. Chemosphere.

[26] Khan NA, Hasan Z, Jhung SH (2013). Adsorptive removal of hazardous materials using metal-organic frameworks (MOFs): A review. J. Hazard. Mater..

[27] Xu W, Wang H, Liu R, Zhao X, Qu J (2011). The mechanism of antimony (III) removal and its reactions on the surfaces of Fe–Mn binary oxide. J. Colloid Interface Sci..

[28] Qi Z, Joshi TP, Liu R, Liu H, Qu J (2017). Synthesis of Ce (III)-doped Fe_3_O_4_ magnetic particles for efficient removal of antimony from aqueous solution. J. Hazard. Mater..

[29] Rangwani S (2018). Adsorptive removal of Sb(V) from water using a mesoporous Zr-based metal–organic framework. Polyhedron.

[30] Qi P (2019). Development of a magnetic core-shell Fe_3_O_4_@TA@UiO-66 microsphere for removal of arsenic (III) and antimony (III) from aqueous solution. J. Hazard. Mater..

[31] Qi Z (2018). Adsorption combined with superconducting high gradient magnetic separation technique used for removal of arsenic and antimony. J. Hazard. Mater..

[32] Du X (2014). Removal of antimony (III) from polluted surface water using a hybrid coagulation–flocculation–ultrafiltration (CF–UF) process. Chem. Eng. J..

[33] Yang S (2018). Controllable NZV-FeZn /reduced graphene oxide hybrid for high-performance supercapacitor electrode. Electrochim. Acta.

[34] Yadav NG (2018). Impact of collected sunlight on NZV-FeZn nanoparticles for photocatalytic application. J. Colloid Interface Sci..

[35] Shen X (2020). Removal of Cr (VI) from acid wastewater by BC/ZnFe_2_O_4_ magnetic nanocomposite via the synergy of absorption photocatalysis. ChemCatChem.

[36] Cao Y (2020). Sb (III) and Sb(V) removal from water by a hydroxyl-intercalated, mechanochemically synthesized Mg-Fe-LDH. Appl. Clay Sci..

[37] Li X, Dou X, Li J (2012). Antimony(V) removal from water by iron-zirconium bimetal oxide: Performance and mechanism. J. Environ. Sci..

[38] Yang C (2021). Efficient removal of Sb(V) in textile waste water through novel amorphous Si-doped Fe oxide composites: Phase composition, stability and adsorption mechanism. Chem. Eng. J..

[39] Guo W (2019). Synthesis of Fe_3_O_4_ magnetic nanoparticles coated with cationic surfactants and their applications in Sb(V) removal from water. Sci. Total Environ..

[40] Chen Y (2021). Composite magnetic photocatalyst Bi24O31Br 10 /NiFe2O4: Hydrothermal preparation, characterization and photocatalytic mechanism. Mater. Sci. Semicond. Process..

[41] Xie Y (2021). Enhanced reactive-oxygen-species generation and photocatalytic efficiency with internal imide structures of different ratio in metal-free perylene-g-C_3_N_4_ semiconductors. Appl. Surf. Sci..

[42] Jones BMF, Maruthamani D, Muthuraj V (2020). Construction of novel n-type semiconductor anchor on 2D honey comb like FeNbO_4_/RGO for visible light drive photocatalytic degradation of Norfloxacin. J. Photochem. Photobiol. A.

[43] Karthikeyan C (2020). Recent advances in semiconductor metal oxides with enhanced methods for solar photocatalytic applications. J. Alloys Compd..

[44] Wu H (2019). Chromium ion removal from raw water by magnetic iron composites and *Shewanella oneidensis* MR-1. Sci. Rep..

[45] Meng F (2018). Temperature dependent photocatalysis of g-C_3_N_4_, TiO_2_ and ZnO: Differences in photoactive mechanism. J. Colloid Interface Sci..

[46] Yang X, Chen S, Zhang R (2014). Utilization of two invasive free-floating aquatic plants (*Pistia stratiotes* and *Eichhornia crassipes*) as sorbents for oil removal. Environ Sci. Pollut. Res. Int..

[47] Lagergren S (1898). About the theory of so-called adsorption of soluble substances. Kungligasvenska Vetenskapsakademienshandlingar.

[48] Freundlich H (1985). Über die adsorption in lösungen. Z. Phys. Chem..

[49] Sips R (1948). On the structure of a catalyst surface. J. Chem. Phys..

[50] Yang X, Guo M, Wu Y, Wu Q, Zhang R (2014). Removal of emulsified oil from water by fruiting bodies of macro-fungus (*Auricularia polytricha*). PLoS ONE.

