# Supplementary material for: Highly efficient removal of Sb(V) from water by franklinite-containing nano-FeZn composites
Source: Sci Rep. 2021 Aug 24;11:17113. doi: 10.1038/s41598-021-95520-7 (PMC8384885; doi:10.1038/s41598-021-95520-7)
Supplement: Supplementary file 1 — Supplementary Information. [file 41598_2021_95520_MOESM1_ESM.docx]

Supplementary materials

**Highly efficient removal of****Sb(V) from water by franklinite-containing nano-Fe-Zn composites**

Huiqing Wu^1^, Qingping Wu^1*^, Jumei Zhang^1^, Qihui Gu^1^, Weipeng Guo^1^, Shun Rong^1^, Yongxiong Zhang^1^, Xianhu Wei^1^, Lei Wei^1^, Ming Sun^1^, Aimei Li^2^, Xinhui Jing^2^

*^1^Guangdong Provincial Key Laboratory of Microbial Safety and Health, State Key Laboratory of Applied Microbiology Southern China, Institute of Microbiology, Guangdong Academy of Sciences, 510070, Guangzhou, China*

*^2^Guangdong Dinghu Mountain Spring Company Limited, Zhaoqing City 526070, Guangdong Province, China*

***Corresponding author.** Email: [wuqp203@163.com](mailto:wuqp203@163.com)

**Optimisation of synthesis**

The process of sample synthesis includes mixing basic synthetic raw materials, ultrasonic treatment at room temperature for 2 hours, dropping alkali solution (NaOH and NaBH4), sealing and standing, centrifugation, water washing and low temperature dry, cooling with nitrogen, sampling, grinding and sieving, weighing, sub packing and vacuumizing, sealing and preservation with N_2_ or Ar. NaOH and NaBH_4_ were used as treatment agents for the synthesis. The specific schemes of conventional synthesis is as follows: 100-mesh rGO or GAC^SP^ (0 or 10 g) was dispersed in degassed water (100 mL), containing 1.1 M FeSO_4_, 0.5 M ZnSO_4_, and 1% PEG-6000, and the dispersion was sonicated at 80 kHz for 120 min. Subsequently, a freshly prepared solution of NaBH_4_ (12.11 g) or NaOH (12.11 g, saturated) in 30:70 (v/v) degassed water: absolute ethanol (NaBH_4_:(Zn^2+^ + Fe^2+^) = 2:1, mol/mol) was added to the dispersion at 50–60 drops min^−1^. The reaction mixture was sealed and allowed to react for 2 h. The solids were separated via centrifugation, rinsed with deionised water, centrifuged two more times, vacuum-dried in an oven at 80–90 °C, and stored in vials under argon for subsequent experiments. 1) The prepared samples were centrifuged at 80–95 °C, dried at a low temperature, removed under nitrogen, ground, passed through a 100-mesh sieve, vacuumized, and stored under nitrogen. 2) Calcination at 300 °C: The sample obtained by the basic process was vacuumized, filled with Ar gas in ceramic appliances after stamping, placed in a muffle furnace and heated to 300 °C (70% heating range) for 2 h, cooled to room temperature, vacuumized, filled with nitrogen, and sealed for preservation. 3) Calcination at 500 °C was performed similar to that at 300 °C for 2 h, and the temperature was decreased to 30 °C before preservation in nitrogen. Supplementary Table S1 shows the Sb removal performances of samples prepared using NaOH and NaBH_4_ methods under various conditions.

The results were as follows. 1) Under identical treatment conditions, the samples synthesised using the NaBH_4_ method had a higher adsorption capacity than those prepared using the NaOH method. 2) The maximum adsorption capacities of the controls exceeded those of samples with GAC^SP^ and rGO as carriers prepared at 95, 300, and 500 °C. However, the yields were higher for carrier-containing samples. 3) After calcination for 2 h at 300–500 °C, the removal efficiency was acceptable, although no enhancement was observed for the CK sample, and calcination had a more pronounced effect in the case of graphene. 4) After calcination at 300–500 °C, the maximum adsorption capacity increased, but the sample yield decreased. Therefore, the NaBH_4_ low-temperature vacuum-drying method was selected to synthesise adsorbents without further calcination. 5) In the chosen process, the performance of the sample obtained using powder-activated special-formula carbon was almost identical to that observed for preliminarily purified graphene (rGO).^2^

**Supplementary Table S1.** Sb removal performances of samples prepared using NaOH and NaBH_4_ methods under various conditions.

| NaOH method | | | | NaBH_4_ method | | | | Loading |
| --- | --- | --- | --- | --- | --- | --- | --- | --- |
| Wt (g)/p  unit | Q1 (<95 °C) | Q2  (300 °C) | Q3  (500 °C) | Wt(g)/p unit | Q1 (<95 °C) | Q2  (300 °C) | Q3  (500 °C) |  |
| 13.83 | 80.21 | 76.66 | 13.42 | 21.45 | 165.49 | 128.57 | 113.96 | No (CK) |
| 12.70 | 97.02 | 79.55 | 33.49 | 18.24 | 163.12 | 128.18 | 78.48 |  |
| 12.52 | 85.55 | 58.16 | 55.14 | 18.06 | 165.49 | 129.19 | 86.08 |  |
| 25.08 | 117.42 | 52.5 | 42.69 | 30.87 | 162.38 | 130.25 | 96.49 | GAC^SP^ |
| 20.62 | 116.05 | 46.54 | 11.14 | 24.42 | 148.41 | 128.19 | 96.75 |  |
| 19.35 | 121.3 | 88.67 | 16.56 | 22.38 | 152.72 | 129.82 | - |  |
| 23.83 | 57.14 | 51.89 | 23.73 | 31.08 | 147.06 | 125.76 | 124.87 | rGO |
| 22.28 | 66.14 | 62.07 | 42.78 | 27.40 | 150.49 | 126.85 | 95.24 |  |
| 21.02 | 62.85 | 85.75 | 16.64 | 26.74 | 141.12 | 125.96 | 75.23 |  |

**Sb removal performances obtained using the oxidation-reduction method at different initial concentrations**

Fe(II):Sb(V) molar ratios of 3:1 and 6:1 were used, and an Fe(II)-free sample was used as a control. The initial concentrations of Sb(V) were set to ~1, 6, 20, 80, and 239.36 mg L^−1^. The reaction mixture was homogenised for 10 min, and pH was adjusted to 10 with 0.1–1 M NaOH to induce flocculation. After proper dilution, the supernatant was analysed by ICP-MS to determine Sb levels (Supplementary Table S2).

**Supplementary Table S2.** Efficiencies of Sb(V) removal by the redox method (*R*%) obtained for different initial Sb concentrations.

| No. | Key points of treatment process | | Ppm | *R*% |
| --- | --- | --- | --- | --- |
|  | Fe(II):Sb(V) | pH |  |  |
| 1 | 6 | 10 | 19.49 | 91.86 |
| 2 | 3 | 10 | 37.01 | 84.54 |
| 3 | 0 | 10 | 209.55 | 12.45 |
| 4 | 0 | natural | **239.36** |  |
| 5 | 6 | 10 | 12.57 | 85.11 |
| 6 | 3 | 10 | 34.32 | 58.38 |
| 7 | 0 | 10 | 59.38 | 27.59 |
| 8 | 0 | natural | **81.83** |  |
| 9 | 6 | 10 | 11.02 | 43.83 |
| 10 | 3 | 10 | 15.47 | 21.15 |
| 11 | 0 | 10 | 18.24 | 7.03 |
| 12 | 0 | natural | **19.62** |  |
| 13 | 6 | 10 | 3.82 | 34.14 |
| 14 | 3 | 10 | 4.04 | 30.34 |
| 15 | 0 | 10 | 4.44 | 23.45 |
| 16 | 0 | natural | **5.80** |  |
| 17 | 6 | 10 | 2.18 | 62.15 |
| 18 | 3 | 10 | 2.34 | 59.38 |
| 19 | 0 | 10 | 2.85 | 50.52 |
| 20 | 0 | natural | **5.76** |  |
| 21 | 6 | 10 | 0.53 | 44.79 |
| 22 | 3 | 10 | 0.59 | 38.54 |
| 23 | 0 | 10 | 0.82 | 14.58 |
| 24 | 0 | natural | **0.96** |  |

The results showed that the pH adjustment method allowed Sb(V) to be removed from water at three different concentrations (high, medium, and low). However, compliance with the drinking water standard was achieved only at a low initial Sb concentration.

**Efficiency of flocculation-based Sb(V) removal using pure FeSO_4_ and ZnSO_4_**

Experiments were performed at an initial Sb(V) concentration of 2 mM, PAC-02 concentration of 20 mg L^−1^, and Fe(II):Sb(V) molar ratios of 1:0–1:6. The mixed Fe(II)-Sb(V) solution was allowed to react for 20 min, and pH was adjusted to 10 with 1.0 M NaOH. The reaction mixture was subjected to centrifugation or flocculation to obtain the supernatant. The concentration of ZnSO_4_ was fixed at 0.02 mM, and Fe(II):Sb(V) molar ratios of 3:1, 2:1, 1:1, 0.5:1, and 0.025:1 were used. After reacting for 20 min, the mixture was pH-adjusted and sampled as above. The Sb content of supernatants was determined by ICP-MS, with the results presented in Supplementary Table S3.

**Supplementary Table S3.** Sb(V) removal performances obtained using pure FeSO_4_ and ZnSO_4_ with PAC as a coagulant.

| No. | Zn^2+^(M) | | Sb(V):Fe(II) (mol/mol) | | ppm | *R*% | Sampling method |
| --- | --- | --- | --- | --- | --- | --- | --- |
| CK1 | | 0 | | 1:0 | 43.16 | 0.00 | Centrifugation (10,000 rpm, 5 min) |
| 1 | |  |  | 1:3 | 26.08 | 39.57 |  |
| 2 | |  |  | 1:4 | 24.44 | 43.37 |  |
| 3 | |  |  | 1:5 | 21.92 | 49.21 |  |
| 4 | |  |  | 1:6 | 18.56 | 57.00 |  |
| 5 | | 0.02 | | 1:0.025 | 28.64 | 33.64 |  |
| CK2 | | 0 | | 1:0 | 43.16 | 0.00 | Flocculation overnight |
| 1’ | | 0 | | 1:3 | 26.08 | 39.57 |  |
| 2’ | |  |  | 1:4 | 24.44 | 43.37 |  |
| 3’ | |  |  | 1:5 | 21.92 | 49.21 |  |
| 4’ | |  |  | 1:6 | 18.56 | 57.00 |  |
| 5’ | | 0.02 | | 1:0.025 | 19.28 | 50.97 |  |

The results showed that more efficient Sb(V) removal was obtained for flocculation with 20 mg L^−1^ PAC-02, as the initial concentration of Sb(V) was set to 2 mM, whereas in the experiment with PAC-02, the CK value greatly decreased after the same treatment. Although treatment with FeSO_4_ alone was effective for Sb(V) removal, the treated water did not meet the national drinking water standard (GB 5749-2006, [Sb] = 0.005 mg L^−1^), and a large Fe(II):Sb(V) molar ratio of 6:1 was used. The presence of a small amount of ZnSO_4_ increased the efficiency of Sb(V) removal by FeSO_4_ and allowed one to decrease its loading and thus prevent the supernatant from turning yellow.

**Sb(V) removal efficiencies of rGO/NZV-FeZn** **and NZV-FeZn**

The Sb(V) removal performances of NZV-FeZn (2019111401) and rGO/NZV-FeZn (2019111402) were tested at a loading of 0.1 g L^−1^ at initial Sb concentrations of 0–140 mg L^−1^. Sb(V)-contaminated water (50 mL) and the adsorbent of choice (5.0 mg) were mixed and allowed to react for 48 h at 28 °C in the dark. Samples taken at set time intervals were centrifuged at 12,600 rpm for 2 min, and the supernatants were appropriately diluted for analysis.

The experimental results (Fig.S1b) show that the removal efficiency of rGO/NZV-FeZn (2019111402) is not as good as that of NZV-FeZn (2019111401) without graphene in the treatment of very low concentration antimony polluted water, but the removal efficiency of pentavalent antimony in high concentration antimony polluted water is on the contrary. Therefore, rGO/NZV-FeZn was selected for continuous Sb(V) removal because of its higher yield and efficiency.

**Sb(V) removal efficiencies of rGO/NZV-FeZn and NZV-FeZn in the case of continuous treatment**

Glass columns (3.5 cm in diameter and 60 cm in height) were packed to a fixed bed depth of 15.0 cm with [1.0 g of GO/NZV-FeZn](mailto:0.3gFe/Zn@graphene) (2019042803) or NZV-FeZn (2019042801), 35 g of K-04 (granular activated carbon for pure water sterilisation), ~1.5 g (3 × 0.5 g) of cotton, and 10.0 g of D101 macroporous resin soaked in ethanol and washed with 200–300 mL of pure water at a flow rate of 6–8 mL min^−1^. Subsequently, a solution containing Sb(V) (initial concentration = 0.61143 mg L^−1^) and auxiliary reagents (10 mg L^−1^ PAC-02 and 0.5 mg L^−1^ PAM) was continuously passed through the columns at a flow rate of 9–10.0 mL min^−1^.

The results (Supplementary Fig. S1) showed that in the case of continuous Sb(V) removal, the level of Sb in the effluent (except for the first 200 mL) was higher for rGO/NZV-FeZn than that for NZV-FeZn. Besides, the Sb concentration in the effluent before the inflection point was lower for rGO/NZV-FeZn than for NZV-FeZn. Considering the yield and Sb removal efficiency during continuous treatment, rGO/NZV-FeZn was selected as the optimal treatment agent. The above behaviour was ascribed to the presence of rGO and was in line with the results of batch experiments, which demonstrated the ability of rGO to adsorb Sb at high concentrations of this contaminant.


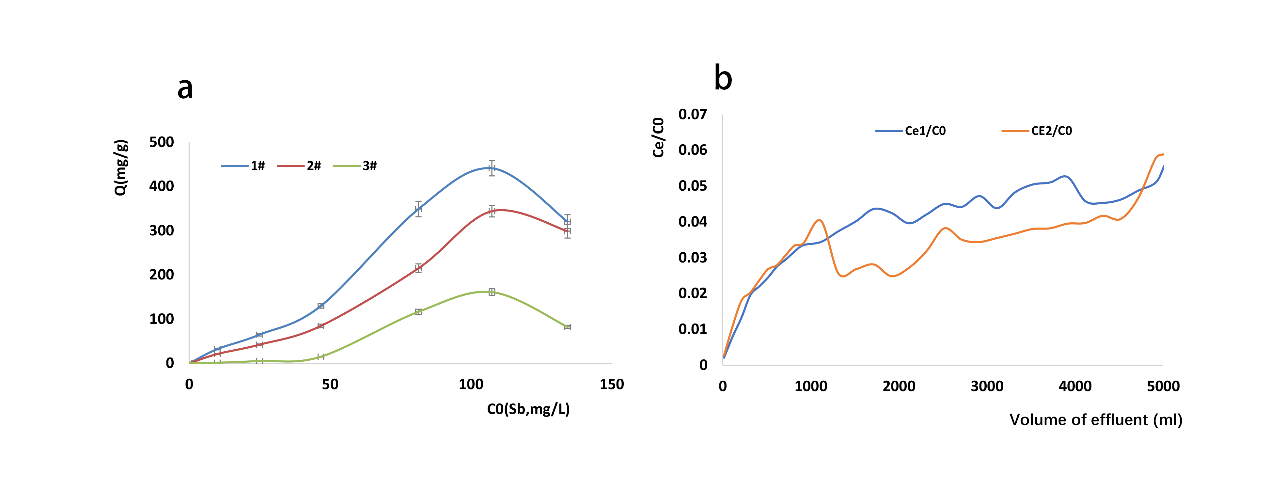


**Supplementary Figure S1.** **a,** Sb(V) removal performances of NZV-FeZn (2019111401) (1#), rGO/NZV-FeZn (2019111402) (2#), and rGO (3#) at a loading of 0.1 g L^−1^ at 28 °C in the dark. **b,** Sb(V) removal performances of columns packed with 1.0 g of NZV-FeZn (2019042801) (1#) and rGO/NZV-FeZn (2019042803) (2#) obtained for continuous treatment at an initial Sb level of 611.43 ppb.

**Regenerability testing of rGO/NZV-FeZn–filled column**

Regenerability testing was performed for a column loaded with 3.5 g of rGO/NZV-FeZn (2018091803) under the conditions described in the main text. The contents of Sb, Fe, Zn, and Al in the effluent were determined for fresh and regenerated columns to probe the reusability (and hence, the practical utility) of the chosen adsorbent (Supplementary Table S4).

The effluents of the first five runs before the inflection point complied with the GB5749-2006 standard (0.005 mg_Sb_ L^−1^), and the corresponding Sb removal efficiency was calculated as 99.38%. However, starting from the second run, the levels of Fe, Zn, and Al in the effluent exceeded those stipulated by GB5749-2006 (0.3, 1.0, and 0.2 mg L^−1^, respectively) in some cases. Considering that Fe, Zn, and Al ions are washed out of the column during multiple runs, the proposed column should be used only once (i.e. should not be regenerated for further use).

**Supplementary Table S4.** Fe, Zn, Al, and Sb contents in effluents collected during six runs.

| No. | *C*_0_ (Sb, mg L^−1^) | *V* (mL) | Al (ppb) | Fe (ppb) | Zn (ppb) | Sb (ppb) | *C*_e_/*C*_0_ (Sb) |
| --- | --- | --- | --- | --- | --- | --- | --- |
| 1 | 0.811 | 15 | 39.137 | 303.242 | 224.01 | 0.322 | 0.0004 |
|  |  | 545 | 253.776 | 31.397 | 99.240 | 0.839 | 0.0010 |
|  |  | 1605 | 212.065 | 6.989 | 20.102 | 4.15 | 0.0051 |
|  |  | 3725 | 223.975 | 6.933 | 4.354 | 4.836 | 0.0060 |
|  |  | 4955 | 184.674 | 2.302 | 1.589 | 23.154 | 0.0285 |
| 2 | 0.811 | 15 | 842.105 | 3977.47 | 6584.72 | 1.204 | 0.0015 |
|  |  | 545 | 1071.117 | 1445.247 | 3441.038 | 4.702 | 0.0058 |
|  |  | 1605 | 744.877 | 327.346 | 1233.240 | 4.953 | 0.0061 |
|  |  | 3725 | 500.654 | 39.989 | 295.470 | 4.993 | 0.0062 |
|  |  | 4955 | 641.960 | 25.981 | 181.676 | 26.343 | 0.0325 |
| 3 | 0.811 | 15 | 101.086 | 1525.047 | 7789.033 | 4.016 | 0.0026 |
|  |  | 545 | 177.337 | 487.056 | 4154.862 | 4.325 | 0.0043 |
|  |  | 1605 | 35.998 | 49.487 | 1636.764 | 4.809 | 0.0039 |
|  |  | 3725 | 24.346 | 10.905 | 687.158 | 16.6 | 0.0063 |
|  |  | 4955 | 6.846 | 4.752 | 326.288 | 62.93 | 0.0089 |
| 4 | 0.8111 | 15 | 153.396 | 619.662 | 601.888 | 6.041 | 0.0039 |
|  |  | 545 | 807.130 | 538.858 | 872.306 | 9.902 | 0.0051 |
|  |  | 1605 | 711.274 | 137.185 | 352.561 | 14.22 | 0.0053 |
|  |  | 3725 | 548.869 | 66.502 | 242.005 | 109.16 | 0.0064 |
|  |  | 4955 | 607.444 | 66.228 | 276.693 | 221 | 0.0315 |
| 5 | 0.8111 | 15 | 228.638 | 337.227 | 230.062 | 4.016 | 0.0050 |
|  |  | 545 | 549.783 | 115.892 | 21.209 | 4.325 | 0.0053 |
|  |  | 1605 | 879.700 | 87.646 | 9.610 | 4.809 | 0.0059 |
|  |  | 3725 | 987.280 | 49.242 | 5.194 | 16.6 | 0.0205 |
|  |  | 4955 | 1214.552 | 77.239 | 6.533 | 62.93 | 0.0776 |
| 6 | 0.8111 | 15 | 692.524 | 88.160 | 12.255 | 6.041 | 0.0074 |
|  |  | 545 | 800.654 | 39.989 | 9.547 | 9.902 | 0.0122 |
|  |  | 1605 | 1169.941 | 33.755 | 8.145 | 14.22 | 0.0175 |
|  |  | 3725 | 1169.941 | 33.755 | 8.145 | 109.16 | 0.1346 |
|  |  | 4955 | 642.185 | 1516.584 | 698.148 | 221 | 0.2725 |

**Fourier transform infrared (FTIR) spectra of the synthesised adsorbents**

The surface chemical structures and compositions of GAC^SP^/NZV-Fe-Zn and rGO/NZV-Fe-Zn were probed by FTIR spectroscopy (Perkin-Elmer Instrument Co. Ltd., USA). For temperature-programmed reduction (Autosorb-1 analyser), the adsorbent (~30 mg) was heated at 473 K in a flow of 5% H_2_/Ar for 1 h and then in pure He for 1 h at the same temperature. The system was cooled to 313 K under He, the atmosphere was changed to 5 vol% H_2_/Ar (30 mL min^−1^), and the catalyst was heated to 1073 K at 10 K min^−1^. The amount of consumed H_2_ was determined using a thermal conductivity detector.

Supplementary Figure S2 presents the FTIR spectra of freshly prepared 100-mesh GAC^SP^/NZV-FeZn and rGO/NZV-FeZn. The broad band at 3375.3–3417.1 cm^−1^ observed in both cases corresponded to the O–H stretching of surface hydroxyl groups, while bands at 1348.6–1354.9 and 1074.1–1100.2 cm^−1^ were assigned to the CH_2_ and C–O groups of long-chain aliphatic components, respectively. The characteristic peaks at 1637.8 and 656.3–685.0 cm^−1^ were mainly related to ester C=O groups and OH groups, respectively. The results indicated the presence of O–H, C=O, C–O, –COOH, and CH_2_ moieties on the surfaces of GAC^SP^/NZV-FeZn and rGO/NZV-FeZn, which means that these surfaces were hydrophilic. The above spectra were similar to that of previously reported GAC^SP^/NZVFe^3^ but featured two additional bands at 610.6–611.3 and 471.2–487.8 cm^−1^ and a shifted (from 2922.4 to 3377.3 cm^−1^) high-wavenumber peak. Thus, FTIR spectra revealed only small differences between active carbon loaded with nano-Fe and Zn and graphene loaded with nano-Fe and Zn.


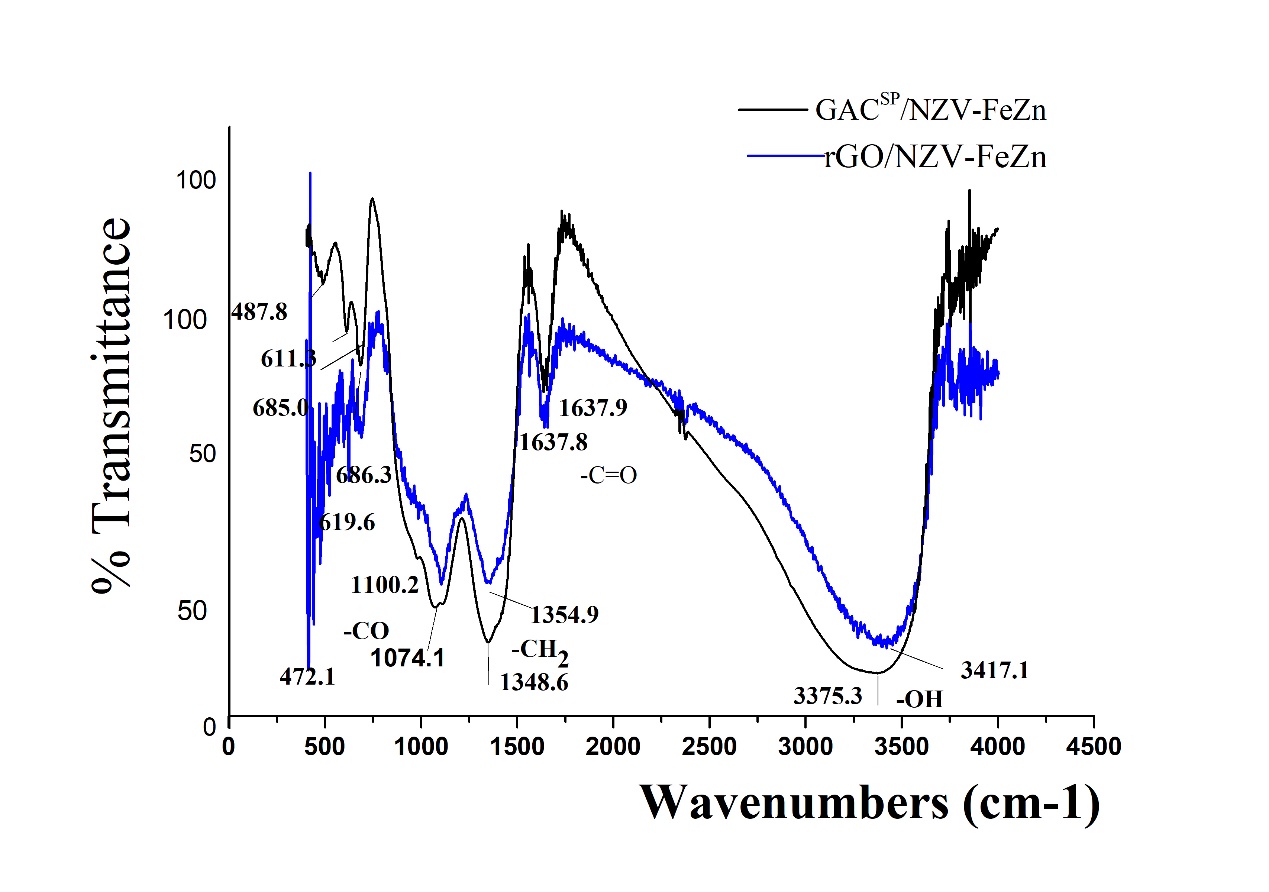


**Supplementary Figure S2.** FTIR spectra of rGO/NZV-FeZn(2018091803) and GAC^SP^/NZV-FeZn (2018091801).

**Magnetic properties of adsorbents**

Magnetic measurements were carried out on a superconducting quantum interference magnetometer at 25 °C (Quantum Design PPMS-9, Quantum Design, Inc., USA). Supplementary Figure S3 demonstrates the room-temperature (23 °C) magnetic properties of NZV-FeZn (1#,2019040501), GAC^SP^/NZV-FeZn (2#, 2018091801), and rGO/NZV-FeZn (3#, 2018091803), revealing that all three samples behaved as typical weakly magnetic materials with zero remanence and zero coercivity. The magnetic hysteresis loops were investigated using a vibrating sample magnetometer at room temperature. Compared to bare NZV-FeZn nanocrystals, GAC^SP^/NZV-FeZn and rGO/NZV-FeZn showed much lower saturation magnetisations because of the presence of non-magnetic graphene nanosheets.


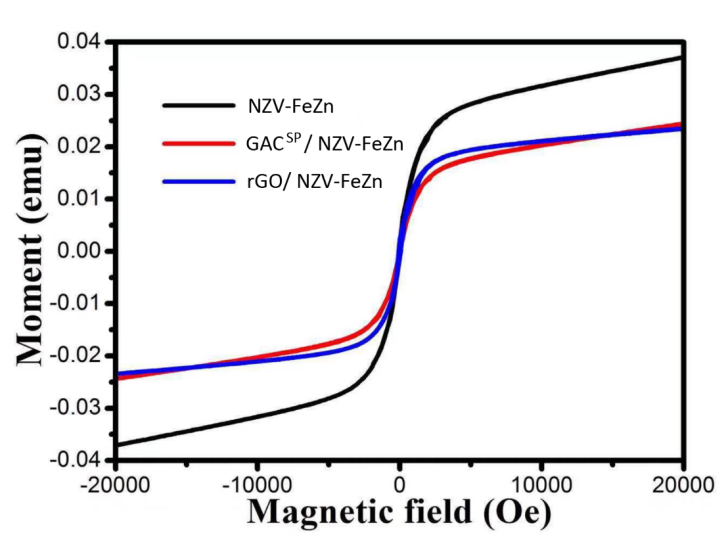


**Supplementary Figure S3.** Magnetic field–dependent magnetic moments of NZV-FeZn, GAC^SP^/NZV-FeZn, and rGO/NZV-FeZn.

Magnetic materials offer the advantage of easy separation after their use for water treatment. The materials synthesised herein were weakly magnetic, particularly those containing non-magnetic carriers, and the above advantage could not be easily exploited. However, in the case of batch treatment, carrier-free NZV-FeZn could be removed using a magnetic field.

**Comparison of the adsorbents synthesised herein with those reported elsewhere**

Supplementary Table S5 compares the adsorbents synthesised herein with those reported elsewhere, revealing that the Sb(V) removal performances of the former were on par with or superior to those of the latter.

**Supplementary Table S5.** Performances of the adsorbents synthesised herein and those reported elsewhere for the removal of Sb.

| First author | Published | Principal features | Ref.  (main text) |
| --- | --- | --- | --- |
| Zhu, J. | 2011 | The best adsorption capacities for both Sb(III) and Sb(V) (151.8 and 472.8 mg g^−1^, respectively) were observed for MIL-101(Fe) and exceeded those of most adsorbents reported previously. | 19 |
| Yao, S. | 2019 | The maximum adsorption capacity of the synthesised adsorbent for Sb(III) at pH 7 was calculated from Langmuir adsorption isotherms in batch experiments as 321.03 mg g^−1^, and the average oxidation percentage reached 95.43% within 1440 min. | 25 |
| Rangwani, S. | 2018 | The overall maximum adsorption capacity equalled 260 mg Sb(OH)_3_ per g of MOF | 30 |
| Qi, Z. | 2017 | A MnFe_2_O_4_-based sorbent could decrease the levels of As or Sb to comply with the US EPA drinking water standard even at a relatively low initial loading of 50.0 μg L^−1^. | 32 |
| Du, X. | 2014 | The optimum FC dosage and solution pH range were 0.4 mM and 7.1–9.0, respectively. Under these conditions, the Sb(III) concentrations in the CF–UF effluent were as low as 1.0–2.0 μg L^−1^, i.e., were significantly lower than the limit stipulated by drinking water standards | 33 |
| Cao, D. | 2019 | The removal of Sb(V) and Co(II) could be effectively achieved by Fe electrocoagulation. At a current density of 5 mA cm^−2^, the concentrations of Sb(V) and Co(II) could be reduced from 60 and 29 mg L^−1^, respectively, to <0.1 mg L^−1^ within 20 min. | 20 |
| This study |  | Under batch treatment conditions, the maximum treatment capacities equalled 543.9 mg g^−1^ for NZV-FeZn (*R*^2^ = 0.951) and 391.1 mg g^−1^ for rGO/NZV-FeZn (*R*^2^ = 0.965). The reaction column constructed with 3.5 g of rGO/NZV-FeZn had a high Sb(V) removal efficiency of 99.38% and good regeneration performance at an initial Sb(V) concentration of 0.8111 mg L^−1^. The Sb content in treated water obtained during the first five runs before the inflection point met the GB5749-2006 requirement. |  |

**REFERENCES**

1. Wu, H. *et al*. Chromium ion removal from raw water by magnetic iron composites and Shewanella oneidensis MR-1. *Sci. Rep.* **9**, 3687 (2019).

2. Liu, Y. *et al*. A facile strategy for preparation of magnetic graphene oxide composites and their potential for environmental adsorption. *Ceram. Int.* **44,** 18571–18577 (2018).

3. Yang, S. H. *et al*. Controllable ZnFe_2_O_4_/reduced graphene oxide hybrid for high-performance supercapacitor electrode. *Electrochim. Acta* **268,** 20–26 (2018).
